# Cell surface-localized CsgF condensate is a gatekeeper in bacterial curli subunit secretion

**DOI:** 10.1038/s41467-023-38089-1

**Published:** 2023-04-26

**Authors:** Hema M. Swasthi, Joseph L. Basalla, Claire E. Dudley, Anthony G. Vecchiarelli, Matthew R. Chapman

**Affiliations:** Department of Molecular, Cellular, and Developmental Biology, University of Michigan, Ann Arbor, MI 48109-1048 USA

**Keywords:** Protein aggregation, Biofilms

## Abstract

Curli are functional amyloids present on the outer membrane of *E. coli*. CsgF is required for the proper assembly of curli. Here, we found that the CsgF phase separates in vitro and that the ability of CsgF variants to phase-separate is tightly correlated with CsgF function during curli biogenesis. Substitution of phenylalanine residues in the CsgF N-terminus both reduced the propensity of CsgF to phase-separate and impaired curli assembly. Exogenous addition of purified CsgF complemented *csgF* ^−^ cells. This exogenous addition assay was used to assess the ability of CsgF variants to complement *csgF* ^*‒*^ cells. CsgF on the cell surface modulated the secretion of CsgA, the curli major subunit, to the cell surface. We also found that the CsgB nucleator protein can form SDS-insoluble aggregates within the dynamic CsgF condensate. We propose that these multicomponent CsgF-B condensates form a nucleation-competent complex that templates CsgA amyloid formation on the cell surface.

## Abstract (web summary)

In this work, the authors show that the phase separation of a cell surface-associated protein called CsgF is critical to mediate the secretion and assembly of the amyloidogenic curli subunits.

## Introduction

Protein misfolding and amyloid deposition are implicated in many neurodegenerative disorders such as Parkinson’s disease and Alzheimer’s disease^1,2^. ‘Functional’ amyloids are distinguished in that the amyloid structure is tightly linked to a desired physiological outcome for the cells^3–5^. Curli fibers are functional amyloids found in the extracellular matrix of enteric bacteria such as *E. coli* and *Salmonella*^6^. The bacterial functional amyloid curli shares biochemical and biophysical properties with disease-associated amyloids^6^. Curli are involved in biofilm formation, cell–cell adhesion, cell–host interaction, and can trigger immune responses in host cells^6,7^. Curli amyloids are composed of a major subunit called CsgA (curli-specific gene) and a minor subunit called CsgB^8^. CsgA amyloid formation at the cell surface is initiated by the CsgB ‘nucleator’ protein^8–10^. Five accessory proteins are necessary to regulate the secretion of curli subunits and to ensure the proper formation of curli on the cell surface. The curli subunits and the accessory proteins are divergently transcribed from the operons *csgDEGF* and *csgBAC*^11–13^. CsgD, the curli master regulator, regulates the expression of all curli-associated proteins^14^. CsgG is a nonameric membrane pore that spans the outer membrane and serves as the channel for the secretion of CsgA, CsgB, and CsgF^15–17^. The binding of CsgE to the periplasmic region of CsgG is essential for substrate selection^16,18^. CsgC is a chaperone-like protein that prevents the aggregation of CsgA in the periplasmic region of bacterial cells^19^. CsgA is secreted to the outer membrane in a soluble unpolymerized form^20^. On the cell surface, CsgB nucleates CsgA amyloid formation^8^. The accessory protein CsgF is essential for the proper anchoring of CsgB onto the cell surface, and without CsgF, curli formation is disrupted^12,21^. Recent structural studies using cryo-EM have revealed that the N-terminus of CsgF arranges on the extracellular part of CsgG^22–24^. However, the mechanism by which CsgF aids the curli assembly is poorly understood.

The classical view on bacterial cell organization is rapidly evolving^25^. A growing body of evidence suggests that biomolecules can localize within bacterial cells^25–27^. Membraneless sub-compartmentalization of biomolecules into “biomolecular condensates” via phase separation (density transition) or phase separation coupled to percolation (networking transition) has emerged as a common theme in eukaryotic cells^28–34^. Advances in microscopy techniques have revealed that the intracellular organization of biomolecules in bacteria could also occur via phase separation^27,35,36^. Multivalent weak interactions between proteins or protein-nucleic acids facilitate the assembly of biomolecular condensates^30,37,38^. Proteins with low-complexity domains, intrinsically disordered regions (IDRs), or prion-like regions are shown to undergo phase separation; however, the role of these domains/regions to mediate phase separation depends on their amino acid composition^39–41^. Biomolecular condensates have properties that are distinct from their surroundings and have been shown to be involved in a wide range of cellular functions^29,42^. For example, concentrating specific reactants in biomolecular condensates can enhance biochemical reactions or suppress reactions by sequestering reactants^37,43^. Many neurodegenerative disease-associated amyloid-forming proteins, such as α-synuclein, huntingtin, tau, and the prion proteins, have been shown to phase-separate and aggregate into amyloids within biomolecular condensates^20,29,30,44–46^.

Cell-surface aggregation of CsgA, the major structural component of curli, is nucleated by CsgB^9^. CsgB adopts a protease-resistant structure on the cell surface^21^. CsgF is the curli accessory protein secreted to the outer membrane that helps CsgB to anchor on the cell surface and to attain nucleator activity^21^. The mechanism by which CsgF promotes the nucleator activity of CsgB is not well understood. In this study, we demonstrate that CsgF readily phase separates in vitro and that aromatic residues in the N-terminal region of CsgF are critical for both its phase separation activity in vitro and curli biogenesis in vivo. We find that CsgF on the cell surface regulates the amount of CsgA that is secreted to the outer membrane. Strikingly, we show that the curli nucleator protein CsgB associates with CsgF droplets and selectively undergoes an amyloid transition while CsgF remains dynamic. Taken together, this study sheds light on the interplay between CsgF and curli subunits during the formation of functional amyloids in bacteria.

## Results

### CsgF forms biomolecular condensates in vitro

**Fig. 1:**
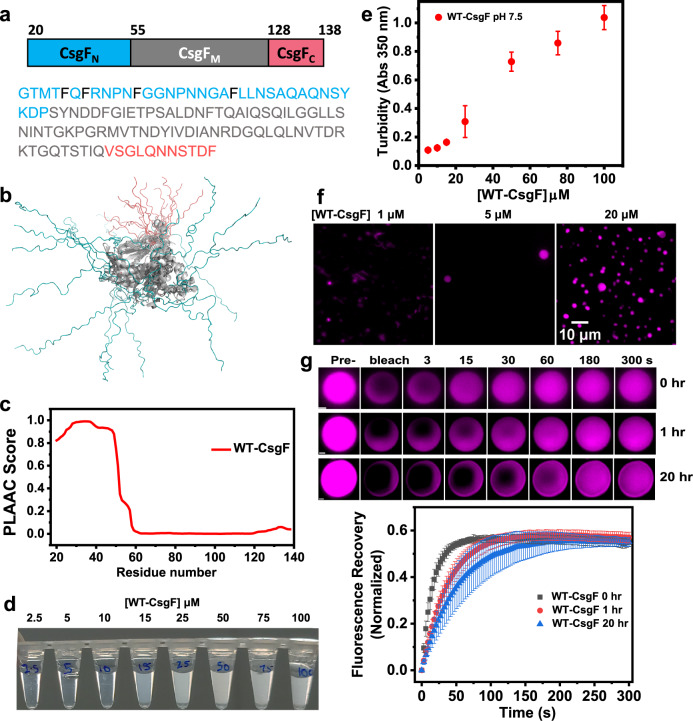
CsgF undergoes phase separation. **a** Schematic representation and amino acid sequence of mature CsgF (CsgF without signal peptide). The N-terminal region (amino acids 20–54) is colored in blue, the middle domain (amino acids 55–127) is shown in gray, and the C-terminal region (amino acids 128–138) is indicated in pink. Phenylalanine residues in the N-terminus are indicated in black. **b** An overlay of 16 conformations of WT-CsgF NMR structure (PDB ID: 5M1U) generated using PyMOL. **c** Prion-like amino acid (PLAAC) analysis on WT-CsgF. **d** Concentration-dependent density change of WT-CsgF in 25 mM potassium phosphate pH 7.5 buffer. **e** Concentration-dependent turbidity change of WT-CsgF, 25 mM potassium phosphate pH 7.5 measured at 350 nm. The data represent mean ± SD, *n* = 4. **f** Fluorescence images of WT-CsgF at 1 µM, 5 µM, and 20 µM in 25 mM potassium phosphate pH 7.5. The unlabeled to labeled WT-CsgF ratio was 50:1. The imaging was carried out three times with similar observations. **g** Time-dependent FRAP measurements on WT-CsgF and quantification of FRAP recovery as a function of time. The scale bar is 1 µm. The data represent mean ± SD, *n* = 3.

Mature CsgF has 119 amino acids, with the first 19 amino acids of CsgF processed during translocation to the periplasm (Fig. 1a). NMR studies on CsgF (PDB ID: 5M1U) have shown that both the N-terminus (CsgF_N_) and C-terminus (CsgF_C_) are unstructured, while the middle region (CsgF_M_) is a mixture of α-helices and β-strands (Fig. 1a, b, and Supplementary Fig. 1a)^47^. CsgF is rich in asparagine (16 residues) and glutamine (11 residues) residues, which is one of the characteristics of prion proteins^48^. Therefore, we subjected wild-type (WT) WT-CsgF to prion-like amino acid composition (PLAAC) analysis^49^. PLAAC predicted that the N-terminus of CsgF (19 to 54) is prion-like (Fig. 1c). Multivalent interactions mediated by low-complexity prion-like domains have been shown to drive phase separation of proteins^39,50^. Therefore, we asked whether CsgF can undergo phase separation. Purified CsgF was incubated at room temperature at pH 7.5 before turbidity was assessed by eye (Fig. 1d) or at 350 nm using a spectrometer (Fig. 1e). Turbidity increased as the WT-CsgF concentration increased from 2.5 to 100 µM (Fig. 1e). Next, lysine residues of WT-CsgF were sparsely labeled with Alexa-633 and mixed with unlabeled protein to a 1:50 (labeled:unlabeled) molar ratio. Fluorescence imaging revealed the formation of phase-separated droplets of WT-CsgF in a concentration-dependent manner (Fig. 1f). The minimum concentration of WT-CsgF required to undergo measurable phase separation was 5 μM (Fig. 1f). WT-CsgF droplets fused to form larger droplets (Supplementary Movie 1). Fluorescence recovery after photobleaching (FRAP) measurements on WT-CsgF condensates revealed ~60% recovery with t_1/2_ = 15 ± 4 s (Fig. 1g). Interestingly, fluorescence recovery of 20-h-old (t_1/2_ = 47 ± 14 s) WT-CsgF droplets was comparable to that of 1-h-old WT-CsgF droplets (t_1/2_ = 36 ± 8 s) (Fig. 1g), suggesting that WT-CsgF condensates do not mature into a more viscous state over time.

We next investigated how pH affected the propensity of WT-CsgF to undergo phase separation. The turbidity of WT-CsgF was measured between a pH range of 4.0–8.5. Below pH 4.5 and above pH 8, there were no apparent turbidity changes, even at the highest concentrations of WT-CsgF (Supplementary Fig. 1b). The pI of WT-CsgF is 4.8 and the predicted net charge of WT-CsgF at pH 4 and 8.5 is +4.3 and −3.9, respectively. We speculated that charge repulsion could prevent WT-CsgF from phase separating at pH 4 and 8.5. Salt can mask the surface charge of proteins; therefore, sodium chloride was added to WT-CsgF at pH 4 to assess changes in its phase separation activity. The addition of 200 mM NaCl induced phase separation of WT-CsgF at pH 4 (Supplementary Fig. 1c). The turbidity of WT-CsgF at pH 4 in the presence of 250 mM NaCl increased as the protein concentration was increased (Supplementary Fig. 1d). At 400 mM NaCl, WT-CsgF formed solid-like aggregates (Supplementary Fig. 1c). Thus, the addition of salt to WT-CsgF at pH 4 could be weakening the ionic interactions and enhancing the non-ionic interactions, which results in phase separation or aggregation of WT-CsgF^51^. Collectively, this data suggested that shielding the surface charge of WT-CsgF promoted its phase separation, which implicates non-ionic interactions as important for WT-CsgF phase separation.

### Phenylalanine residues in the N-terminus modulate CsgF phase separation

**Fig. 2:**
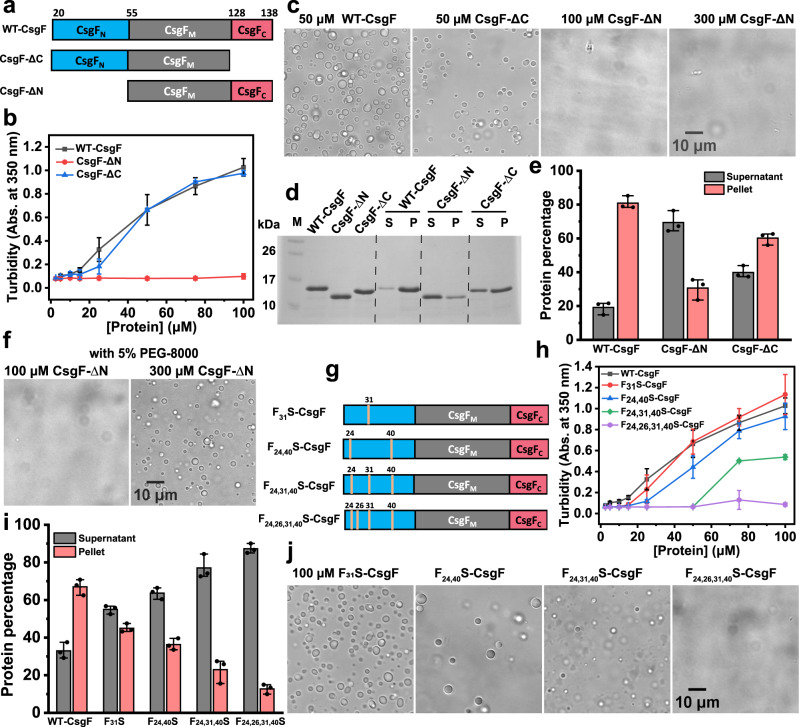
Phenylalanine residues in the N-terminus modulate phase separation of CsgF. **a** Schematic representation of CsgF-∆N and CsgF-∆C used in the study. **b** Turbidity measurements of WT-CsgF, CsgF-ΔN, and CsgF-ΔC in 25 mM potassium phosphate pH 7.5. The data represent mean ± SD, *n* = 3. **c** Images of WT-CsgF (50 µM), CsgF-ΔC (50 µM), and CsgF-ΔN (100 µM and 300 µM) in 25 mM potassium phosphate pH 7.5. **d** Coomassie-stained SDS-PAGE on the supernatant and pellet samples of 50 µM WT-CsgF, CsgF-∆N, and CsgF-∆C after centrifugation. M: Molecular weight markers, S: Supernatant, P: Pellet. **e** SDS-PAGE shown in (**d**) was quantified. The data represent mean ± SD, *n* = 3. **f** DIC images of 100 µM and 300 µM CsgF-∆N in the presence of 5% PEG-8000 (Polyethylene Glycol-8000). **g** The phenylalanine residues that are mutated in the N-terminal region are marked as yellow lines. **h** Turbidity measurements on phenylalanine mutants of CsgF. The data represent mean ± SD, *n* = 3. **i** Sedimentation assay on the phenylalanine mutants of CsgF was quantified from Coomassie-stained SDS-PAGE. The data represent mean ± SD, *n* = 3. **j** DIC images of 100 µM CsgF phenylalanine mutants in 25 mM potassium phosphate pH 7.5. The imaging was performed three times with similar observations (**c**, **f**, **j**).

Structural elucidation of WT-CsgF using NMR has shown that both the N- and C-termini are unstructured (Fig. 1b)^47^. We made N- and C-terminal truncation mutants of CsgF to determine if either IDR was necessary for its phase-separation activity (Fig. 2a). The turbidity of CsgF-ΔC samples increased as the protein concentration increased (Fig. 2b), and imaging confirmed condensate formation (Fig. 2c). CsgF-∆C droplets fused at a similar rate as WT-CsgF (Supplementary Movie 2). CsgF-ΔN, on the other hand, did not show concentration-dependent turbidity changes (Fig. 2b) or visible droplet formation, even at significantly higher protein concentrations (300 μM) (Fig. 2c). WT-CsgF and its variants (20 and 50 µM) were centrifuged and the supernatant and pellet fractions were subjected to SDS-PAGE to quantify proteins in the dilute and condensed phases (Supplementary Fig. 2a and Fig. 2d). The amount of CsgF-∆C in the pellet fraction was reduced by ~20% relative to WT-CsgF, while most of the CsgF-∆N remained in the supernatant (Fig. 2e). Therefore, both the N- and C-terminal disordered regions of CsgF contribute to its phase-separation activity. However, the N-terminus appears to be necessary for the phase-separation activity of WT-CsgF, while the C-terminus is dispensable, at least at relatively high protein concentrations. The phase separation of CsgF-∆N was monitored in the presence of a crowding agent to elucidate whether WT-CsgF can phase-separate without the disordered N-terminus. Phase separation was not observed upon the addition of 5% PEG-8000 to 100 µM CsgF-∆N (Fig. 2f). However, 5% crowding agent-induced phase separation of 300 µM CsgF-∆N (Fig. 2f), which suggested that WT-CsgF can phase-separate even in the absence of the N-terminal IDR. Thus, increasing the local concentration of CsgF-∆N with a crowding agent promoted its phase separation. However, WT-CsgF phase-separated at a concentration of ~5 µM while CsgF-∆N required an approximately 60-fold increase in the protein concentration and the addition of PEG-800 to phase-separate. The N-terminal region might be bringing WT-CsgF together to increase the effective protein concentration and could also be involved in weak multivalent interactions.

Studies have demonstrated that aromatic residues in IDRs determine the phase behavior of proteins^52–54^. WT-CsgF has a total of seven phenylalanine and three tyrosine residues, with four phenylalanine residues and one tyrosine residue in the N-terminal region (Fig. 1a). We created serine substitutions of the phenylalanine residues located in the CsgF N-terminus. Phenylalanine to serine mutants were named F_X_S-CsgF (X = the position where the original phenylalanine was present) (Fig. 2g). To measure how the phase-separation activity of WT-CsgF varies with changes to the number of phenylalanine residues, we first used a turbidity assay. Turbidity measurements indicated that as the number of phenylalanine residues decreased, there was a corresponding reduction in WT-CsgF phase separation (Fig. 2h). F_24,26,31,40_S-CsgF did not show any measurable turbidity (Fig. 2h). Consistently, sedimentation assays performed on 20 µM F_X_S-CsgF mutants revealed a progressive reduction in the amount of protein in the pellet fraction with the decrease of phenylalanine residues (Supplementary Fig. 2b and Fig. 2i). We imaged the phenylalanine to serine mutants at 50 and 100 µM concentrations and found that F_31_S and F_24,40_S-CsgF readily phase-separated at these concentrations (Supplementary Fig. 2c and Fig. 2j). F_24,31,40_S-CsgF, on the other hand, only phase-separated at 100 µM. F_24,26,31,40_S-CsgF showed no phase-separation activity at either of the protein concentrations tested (Supplementary Fig. 2c and Fig. 2i). The sedimentation assays, turbidity measurements, and microscopic imaging suggested that the phenylalanine residues in the N-terminus are required to promote phase separation of WT-CsgF.

### The N-terminus is essential for the secretion of CsgF to the outer membrane

**Fig. 3:**
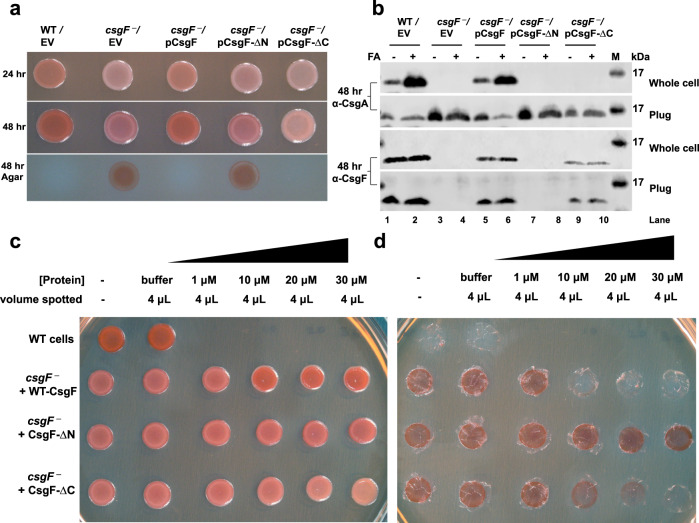
Exogenous CsgF complements *csgF*^*‒*^ cells. **a** Cells were grown on Congo red (CR) indicator plate to assess the phenotypes of WT/Empty vector (MC4100/pLR1), *csgF*^*‒*^/Empty vector (MHR592/pLR1), *csgF*^*‒*^/pCsgF (MHR592/pLR73), *csgF*^*‒*^/pCsgF-ΔN (MHR592/pCsgF-ΔN), and *csgF*^*‒*^/pCsgF-ΔC (MHR592/pCsgF-ΔC), and after 24 and 48 h and agar plate after removing bacterial cells. EV: Empty Vector. **b** Western blots on whole cells and plugs after 48 h of growth on YESCA (Yeast Extract Casamino acids) plates. The samples for blots were prepared either in 2X SDS-PAGE running buffer directly or first being treated with formic acid (FA) before being run on SDS-PAGE, transferred to a membrane, and probed for CsgA and CsgF. Western blotting was performed three times with similar observations. M: Molecular weight markers, FA: formic acid. **c** *csgF*^*‒*^ cells complemented using exogenously added protein. Protein stocks (1, 10, 20, and 30 µM) were made in buffer (25 mM Tris pH 9). Four µL (1, 10, 20, and 30 µM) of protein was spotted on the Congo red YESCA plate and when the protein sample was dried, cells were spotted and allowed to grow for 48 h at 26 °C. **d** Agar plate after removing the bacterial cells shown in (**c**). The complementation assays were performed three times with similar observations (**a**, **c**, **d**).

CsgF assists CsgB in forming a functional curli nucleator complex on the bacterial surface^21^. In the absence of CsgF, cells fail to assemble as much curli on the cell surface as wild-type (WT)^12,21^. We set out to understand the role of the N- and C-terminal IDRs of CsgF by assaying the ability of CsgF-ΔN and CsgF-ΔC variants to complement WT-CsgF function in vivo. Curli-producing *E. coli* cells MC4100 (WT/empty vector (EV)) stain red on Congo red (CR) indicator plates, and the *csgF*^*‒*^/EV cells exhibit a pink color phenotype (Fig. 3a)^12,21^. When *csgF*^*‒*^/EV cells were removed from CR plates after 48 h of growth, the underlying agar was red (Fig. 3a)^21^. The red coloration underneath the *csgF*^*‒*^*/*EV cells is due to secreted curli subunits that assemble into CR-binding polymers but did not attach to the cells^21^. The *csgF*^*‒*^ strain was complemented by the pCsgF plasmid (Fig. 3a). However, when *csgF*^*‒*^ cells harboring pCsgF-ΔN were grown under curli-expressing conditions, the phenotype matched the *csgF*^*‒*^ strain where the underlying agar was stained red. Interestingly, the *csgF*^*‒*^/ pCsgF-ΔC cells remained white even after 48 h, and when the cells were scraped off, there was no red staining on the agar (Fig. 3a). Thus, failure of CsgF-ΔN and CsgF-ΔC to complement *csgF*^*‒*^ cells indicated that both N- and C-terminal regions of CsgF are necessary for the function of WT-CsgF.

To better understand how the N- and C-terminal domains contribute to WT-CsgF function in vivo, *csgF*^*‒*^ strains harboring pCsgF-ΔN and pCsgF-ΔC were characterized. Curli-producing WT cells and *csgF*^*‒*^/pCsgF cells need mild formic acid treatment to monomerize the cell-surface polymerized CsgA^12,21^. CsgA that is secreted by curli-forming cells is assembled as amyloid fibers on the outer membrane; thus, there would not be much SDS-soluble CsgA in the agar plug. In *csgF*^*‒*^ strains, CsgA fails to polymerize on the cell surface, allowing unpolymerized CsgA to secrete away from the cell surface^21^. Therefore, the agar plug underneath *csgF*^*‒*^/EV cells have SDS-soluble CsgA. Western blot analysis on whole cells and the plug was performed to characterize the nature of CsgA in cells that were expressing CsgF variants. Without formic acid treatment, very little monomeric CsgA was detectable in the WT/EV or *csgF*^*‒*^/pCsgF cells that were collected from YESCA plates after 48 h of growth (Fig. 3b, top). Upon treating the WT/EV and *csgF*^*‒*^/pCsgF cells with formic acid, there was a dramatic increase in the amount of CsgA in the whole cell blot, suggesting that CsgA was in an SDS-insoluble amyloid form (Fig. 3b). On the contrary, for *csgF*^*‒*^/EV cells, CsgA was found in the plugs, and formic acid-treated and non-treated plugs had a comparable amount of CsgA (Fig. 3b). Similarly, *csgF*^*‒*^ cells with pCsgF-ΔN and pCsgF-ΔC, CsgA was neither detected in formic acid-treated nor in non-treated whole cells. Both in pCsgF-ΔN and pCsgF-ΔC containing strains, CsgA was found in the agar plugs. We next probed for CsgF (Fig. 3b, bottom). WT/EV and *csgF*^*‒*^/pCsgF cells showed the presence of WT-CsgF both in the whole cells and plug. However, *csgF*^*‒*^/pCsgF-ΔN did not have detectable bands in either the whole cell or plug samples when stained with CsgF antibody (Fig. 3b, lanes 7 and 8). *csgF*^*‒*^ cells with pCsgF-ΔC were detected by CsgF antibody both in the whole cell and plug samples. To test whether the CsgF antibody binds to CsgF-ΔN, we probed the antibody against purified proteins. CsgF antibody staining of CsgF-∆N matched with WT-CsgF but the CsgF antibody exhibited slightly less binding to CsgF-∆C (Supplementary Fig. 3a). Thus, the absence of CsgF-ΔN in the whole cell or plug was not because of the inability of CsgF antibody to detect CsgF-ΔN variants as the CsgF antibody exhibited similar affinity towards WT-CsgF and CsgF-∆N (Supplementary Fig. 3a). The absence of CsgF-∆N both in the whole cell and plug suggested that CsgF-∆N is unstable and not secreted to the cell surface.

### Phenylalanine residues are critical for curli assembly

Since CsgF is a cell surface protein, we hypothesized that exogenous WT-CsgF would complement *csgF*^*‒*^ cells. To test this, *csgF*^*‒*^ cells were spotted on a CR-YESCA plate that had been supplemented with purified WT-CsgF. After 48 h of growth, the *csgF*^*‒*^ cells appeared pink, but as the concentration of purified WT-CsgF that was added to the plate increased, the cells stained a deeper red (Fig. 3c). Similarly, there was a decrease in the red staining on the CR plate as the concentration of exogenously added WT-CsgF increased (Fig. 3d). Thus, exogenous WT-CsgF partially complemented *csgF*^*‒*^ cells. When CsgF-ΔN protein was added to the *csgF*^*‒*^ cells, the phenotype was similar to *csgF*^*‒*^ cells even at the highest protein concentration tested (1.12 µg). *csgF*^*‒*^ cells grown in the presence of 1.54 µg of CsgF-ΔC variant exhibited a white phenotype, and the red coloration on the plate decreased with an increase in protein concentration (Fig. 3c, d). Thus, the phenotypic features exhibited by *csgF*^*‒*^/pCsgF, *csgF*^*‒*^/pCsgF-∆N, and *csgF*^*‒*^/pCsgF-∆C were recapitulated using endogenous WT-CsgF or its variants. The absence of CsgF-∆N in the whole cell and plug, as well as the phenotypic similarity of *csgF*^*‒*^/pCsgF-∆N and *csgF*^*‒*^, suggested that the N-terminal region of CsgF is required for the function of protein on the cell surface. The failure of endogenous and exogenous CsgF-∆N and CsgF-∆C to complement *csgF*^*‒*^ cells indicated that both the N-terminal and C-terminal regions of CsgF are essential for the function of the protein. The N-terminus of CsgF has a key role to play both during CsgF translocation across the outer membrane and on the cell surface during curli biogenesis.

**Fig. 4:**
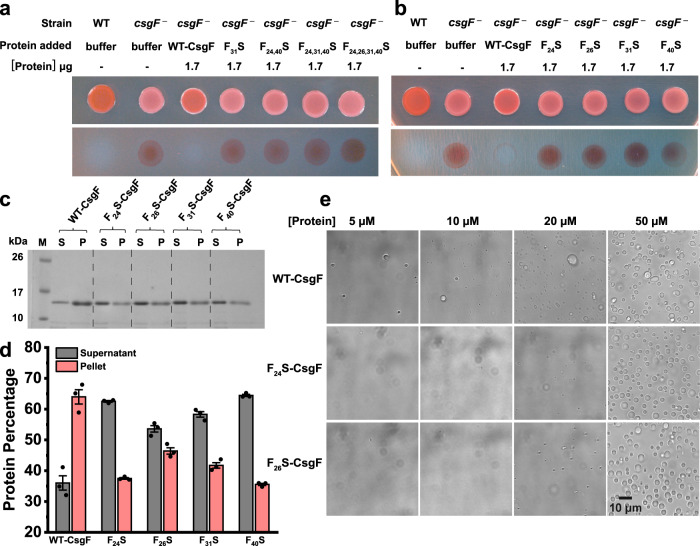
Single phenylalanine mutation impairs curli assembly and CsgF phase separation. **a**, **b** Exogenous complementation of *csgF*^*‒*^ cells with phenylalanine mutants. **c** Coomassie-stained SDS-PAGE on the supernatant and pellet samples of single phenylalanine mutants after sedimentation. M: Molecular weight markers, S: Supernatant, P: Pellet. **d** Quantification of SDS-PAGE shown in (**c**). The data represent mean ± SD. **e** DIC images of 5, 10, 20, and 50 µM of WT-CsgF, F_24_S-CsgF, and F_26_S-CsgF in 25 mM potassium phosphate pH 7.5. The imaging was conducted three times with similar observations.

Next, we focused on elucidating the ability of phenylalanine mutants (Fig. 2g) to complement *csgF*^*‒*^ cells. We used the exogenous complementation method to assess the complementation ability of the phenylalanine mutants. None of the phenylalanine mutants complemented *csgF*^*‒*^ cells (Fig. 4a). Intriguingly, a single F31S mutation was enough to impair the function of WT-CsgF and curli assembly. We also examined single phenylalanine mutation at 24, 26, and 40 positions. CR assay revealed that all the single phenylalanine mutations failed to complement *csgF*^*‒*^ cells (Fig. 4b). Phase-separation activity was assessed to see whether single phenylalanine mutation affected the phase-separation propensity of WT-CsgF. Then, 20 µM samples were sedimented by centrifugation and analyzed on SDS-PAGE (Fig. 4c, d). There was a reduction in the amount of protein present in the pellet fraction of all single F_x_S mutants compared to the WT-CsgF. Imaging of the single phenylalanine mutants suggested all the single phenylalanine mutants phase-separated ~20 µM whereas WT-CsgF phase separates ~ 5 µM (Fig. 4e and Supplementary Fig. 3b). The CR plate assay, together with sedimentation and imaging, suggested that the phenylalanine residues in the CsgF N-terminus are critical for WT-CsgF function and phase separation. Additionally, sequence alignment of CsgF homologs indicated the presence of at least three phenylalanine/tyrosine residues in the N-terminus, and AlphaFold predictions suggested that the N-terminal regions of CsgF homologs were disordered or partially α-helical (Supplementary Fig. 4a, b).

### Surface-localized CsgF regulates CsgA secretion

**Fig. 5:**
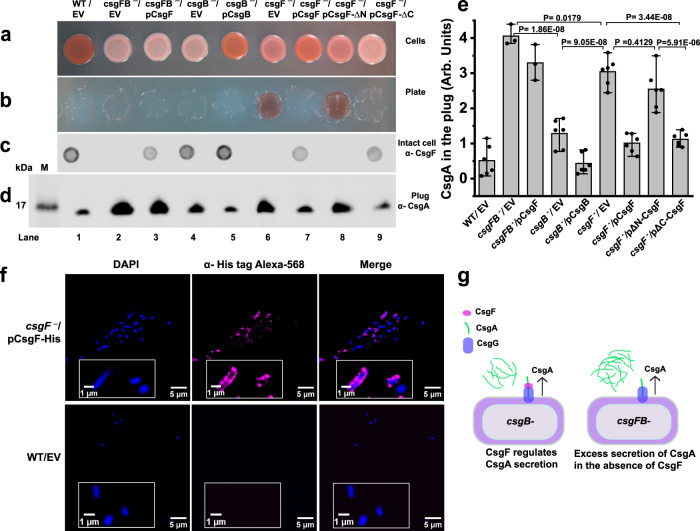
Cell surface-associated CsgF regulates CsgA secretion. **a** Phenotypes of 48 h grown WT/Empty vector (MC4100/pLR1), *csgFB* ^*‒*^/Empty vector (*csgFB* ^*‒*^/pLR2), *csgFB* ^*‒*^/pCsgF (*csgFB* ^*‒*^/pLR73), *csgB* ^*‒*^/Empty vector (MHR261/pLR2) *csgB* ^*‒*^/pCsgB (MHR261/pLR8), *csgF* ^*‒*^/Empty vector (MHR592/pLR1), *csgF* ^*‒*^/pCsgF (MHR592/pLR73), *csgF* ^*‒*^/pCsgF-ΔN (MHR592/pCsgF-ΔN), and *csgF* ^*‒*^/pCsgF-ΔC (MHR592/pCsgF-ΔC) cells on a Congo red YESCA plate. EV: Empty vector. **b** Agar plate after removing the cells shown in (**a**). **c** The dot blot was performed using CsgF antibody on intact cells to assess the presence of surface-associated CsgF. **d** Western blot on agar plugs that were collected from under the cells after they had grown for 48 h. The experiments were performed three times with similar observations in (**a**–**d**). **e** The western blot shown in (**d**) was quantified using Fiji/ImageJ. The data represent mean ± SD, *n* = 3. *P*-values were determined by one-way ANOVA with the Tukey test. **f** Immunofluorescence images of *csgF* ^*‒*^/pCsgF-His (MHR592/pCsgF-His). The cells were fixed with formaldehyde and probed with His-tag antibody and then with Alexa-568 secondary antibody. WT/Empty vector (MC4100/pTrc99A) was used as a control. EV: Empty vector. The imaging was performed three with similar observations. **g** A model to show the presence of surface-associated CsgF regulates CsgA secretion.

The unpolymerized soluble CsgA that is secreted away from a *csgB*^*‒*^ strain (CsgA^+^ donor) can assemble on the surface of *csgF*^*‒*^ strain (CsgB^+^ recipient) by the process of interbacterial complementation^55^. The *csgFB*^*‒*^ double mutant cells are better CsgA donors compared to *csgB*^*‒*^ cells^12^, and why *csgFB*^*‒*^ cells are better CsgA donors is unknown. We performed a CR assay on CsgA donors *csgFB*^*‒*^/EV, *csgFB*^*‒*^*/*pCsgF, *csgB*^*‒*^/EV, along with *csgF*^*‒*^ and its variants. The *csgF*^*‒*^/EV and *csgF*^*‒*^/pCsgF-ΔN cells were pink on CR indicator plates, while all the other strains remained white or unstained (Fig. 5a, b). Only *csgF*^*‒*^/EV and *csgF*^*‒*^/pCsgF-ΔN stained red underneath the cells. To see the presence of CsgF on the bacterial surface, we carried out intact cell dot blot assays. Cells grown on YESCA plates for 48 h were normalized to 1 OD_600_ and spotted on nitrocellulose membrane and probed for CsgF. As expected, WT/EV, *csgFB*^*‒*^/pCsgF, *csgB*^*‒*^/EV, *csgB*^*‒*^/pCsgB, *csgF*^*‒*^/pCsgF, and *csgF*^*‒*^/pCsgF-∆C showed the presence of surface-associated CsgF (Fig. 5c). Next, we performed a plug assay without formic acid to determine the amount of soluble CsgA that was being secreted by these non-curli-producing cells. As a loading control, we ran the samples shown in Fig. 5d on SDS-PAGE and stained them with Coomassie blue (Supplementary Fig. 3c). Interestingly, we observed differences in the amount of CsgA in the plug for the non-curli-producing cells (Fig. 5d, e). The amount of CsgA secreted by the *csgFB*^*‒*^/EV double mutant cells was higher compared to other non-curli-producing cells*. csgB*^*‒*^/EV cells secreted ~2.5-fold less CsgA relative to the *csgFB*^*‒*^/EV cells (Fig. 5e). Similarly, *csgF*^*‒*^/pCsgF-∆C secreted less CsgA protein than *csgFB*^*‒*^/EV cells. Thus, the presence of cell surface CsgF in the non-curli-producing cells governed the CsgA secretion. Next, we performed immunofluorescence microscopy to visualize cell surface-associated CsgF. Immunofluorescence imaging of *csgF*^*‒*^/pCsgF-His revealed that WT-CsgF localized as puncta on the cell surface (Fig. 5f). The plug and immunofluorescence assays, together with the intact cell dot blot, indicated that WT-CsgF localizes to the bacterial surface and is a regulator of CsgA secretion in the non-curli-producing cells (Fig. 5g).

### CsgB incorporates into phase-separated droplets of CsgF

**Fig. 6:**
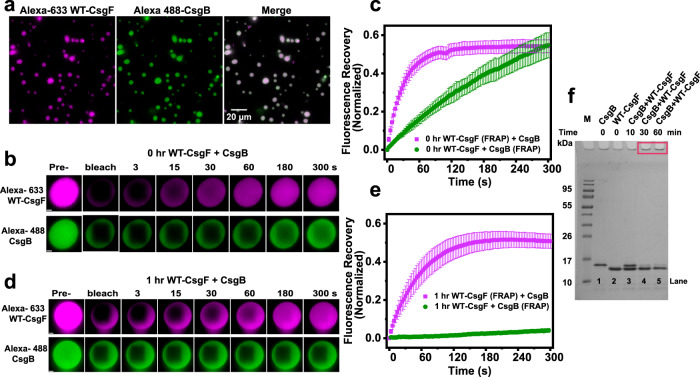
CsgB localizes in CsgF condensates. **a** Fluorescence images of Alexa-633-WT-CsgF (20 µM) and Alexa-488-CsgB (20 µM). Unlabeled to labeled protein molar ratio used was 50:1. The imaging was carried out three times with similar observations. **b** FRAP on WT-CsgF and CsgB immediately after mixing. Scale bar: 1 µm. **c** Quantification of fluorescence recovery of CsgF and CsgB for 0 h sample. The data represent mean ± SD, *n* = 3. **d** FRAP measurements on WT-CsgF and CsgB 1 h after mixing. Scale bar: 1 µm. **e** Quantification of fluorescence recovery of WT-CsgF and CsgB after 1 h incubation. The data represent mean ± SD, *n* = 3. **f** Coomassie-stained SDS-PAGE of WT-CsgF (20 µM) and CsgB (20 µM) alone or together. The SDS-PAGE wells of 30 min and 60 min WT-CsgF (20 µM) +CsgB (20 µM) are highlighted in the red box. M: Molecular weight markers. SDS-PAGE was performed three times with similar observations.

Curli subunits are secreted to the cell surface via the CsgG membrane channel. Cryo-EM structural analysis has shown that the N-terminal region of CsgF binds to the CsgG channel^22–24^. CsgB and CsgA could potentially interact with the CsgG-CsgF complex while traversing to the cell surface. In the absence of WT-CsgF, CsgB fails to anchor on the cell surface and nucleate CsgA polymerization^21^. We asked whether CsgB could interact with the WT-CsgF condensates we identified here. When CsgF (20 µM) condensates were added to CsgB (20 µM), both proteins colocalized (Fig. 6a). However, CsgB alone did not undergo phase separation under WT-CsgF condensate-forming conditions (Supplementary Fig. 5a). The C-terminal region (R5 repeat) of CsgB is positively charged and a CsgB truncation mutant that is missing R5 (CsgB∆R5) does not aggregate into amyloid fibers as readily as WT-CsgB^10,56^. CsgB∆R5 still sequestered into WT-CsgF phase-separated droplets (Supplementary Fig. 5b). CsgB shares ~30% sequence identity with CsgA^9^. To see whether the interaction between CsgB and WT-CsgF was specific, we also performed fluorescence imaging of WT-CsgF condensates in the presence of CsgA. Unlike CsgB, CsgA was not recruited into CsgF condensates (Supplementary Fig. 5c). To test the possibility that CsgB was promiscuously recruited to phase-separated condensates, we added mixed CsgB condensates made of McdB (Maintenance of Carboxysome Distribution), a cyanobacterial protein. CsgB failed to sequester in the McdB droplets (Supplementary Fig. 5d) in the way that it did with CsgF condensates (Supplementary Fig. 5d). Next, to elucidate the nature of CsgB in the phase-separated droplets of WT-CsgF, we carried out FRAP measurements. CsgB recruited in the WT-CsgF droplets exhibited a slow and linear fluorescence recovery immediately after mixing (Fig. 6b, c). The half-time recovered from the fluorescence recovery kinetics of CsgF immediately after mixing with CsgB and after an hour were 32 ± 6 s, and 56 ± 15 s, respectively (Fig. 6d, e). CsgB, on the other hand, failed to show any notable fluorescence recovery after an hour. SDS-PAGE was performed to further understand the nature of CsgB in the presence of WT-CsgF. Samples collected after 10 min contained SDS-soluble CsgB (Fig. 6f). However, after 30 and 60 min, the samples had very little soluble CsgB and most of the CsgB was stuck in the SDS-PAGE wells (Fig. 6f). The SDS-PAGE data suggested the formation of SDS-insoluble higher order CsgB aggregates in the presence of phase-separated CsgF droplets. It is noteworthy that the material state of CsgB selectively transitioned from a dynamic to an amyloid state in the multicomponent droplets, while WT-CsgF did not undergo notable changes. Fluorescent imaging of curli subunits with CsgF indicated that the interaction between CsgB and WT-CsgF droplets is specific and that CsgB undergoes amyloid transition within the dynamic CsgF condensates.

## Discussion

Curli are bacterial functional amyloids found on the surface of enteric bacteria such as *E. coli* and *Salmonella* spp^6^. Curli are the major protein component of enteric bacterial biofilm. The formation of many disease-causing amyloids is sporadic; however, curli production is well-orchestrated. CsgA protein, the major structural component of curli, is secreted to the cell surface in an unstructured state^8^. CsgF is a curli accessory protein that assists the CsgB nucleator in providing a folding template that enables CsgA amyloid aggregation at the cell surface^21^. Both CsgA and CsgB are secreted through the CsgG-CsgF outer membrane pore^57^. NMR studies on CsgF have shown that the N-terminus and the C-terminus of CsgF are disordered (Fig. 1b)^47^. The conformational plasticity of IDRs makes them a better candidate for multiple functions^58^. A large body of evidence has suggested that multivalent weak interactions involving proteins with disordered regions or low-complexity domains mediate phase separation^39,50,52^. The presence of a prion-like low-complexity region in CsgF led us to hypothesize that CsgF might have the propensity to undergo phase separation (Fig. 1c). CsgF readily formed phase-separated droplets in a concentration-dependent manner without any crowding agents (Fig. 1d–f). The N-terminus disordered region had a greater impact compared to the C-terminus in mediating CsgF phase separation (Fig. 2b–e). CsgF function in vivo was affected by the deletion of either the N- or C-terminus (Fig. 3a–d). It has been shown for CsgA that its N-terminus mediates CsgA–CsgG interaction at the periplasm, which allows translocation of CsgA to the cell surface^23^. CsgA devoid of the N-terminal region (N-22 domain) is unstable in the periplasm^57^. CsgF also secretes to the outer membrane via CsgG^21^. The absence of CsgF-∆N both in the whole cell and plug hinted that the CsgF N-terminus is essential for CsgG recognition and secretion. Thus, CsgF (without N-terminus) that are accumulated in the periplasm are susceptible to proteolytic degradation. Congo red, exogenous complementation, and Western blot assays suggested that the N-terminal region is essential for the secretion of CsgF across the outer membrane, for the function of CsgF on the cell surface, and for the phase separation of CsgF.

Phase separation of prion-like domains is often described using a stickers-and-spacers model^39,52,59^. Stickers are the amino acid residues that promote intra- and inter-molecular reversible networks, whereas spacers are the residues that are interspersed between stickers^60^. Studies have shown that aromatic residues (Y/F) in the prion-like region act as stickers^52,53^. The number and patterning of aromatic residues (valence) govern the phase behavior of prion-like domains^52^. When phenylalanine residues in the N-terminus of CsgF were replaced with serine, a chemically neutral spacer, the phase-separation activity of CsgF and curli biogenesis were both impaired (Figs. 2g–j, 4, and Supplementary Fig. 3b). A reduction in the propensity of CsgF to undergo phase separation with a decreasing number of phenylalanine residues indicated the importance of aromatic residues in modulating the phase-separation activity of CsgF (Fig. 2g–j). The N-terminus of CsgF embeds on top of the CsgG opening at a 9:9 ratio^22–24^, and single point mutations of phenylalanine residues at 24, 26, and 31 have shown to disrupt the CsgF interaction with CsgG^23^. Our phenylalanine mutant proteins failed to complement *csgF*^*‒*^ cells when added exogenously (Fig. 4a, b). The interactions of phenylalanine residues in the N-terminal region are essential for anchoring CsgF onto CsgG. The nonameric CsgG membrane channel could be a molecular crowder on the cell surface to bring CsgF molecules close enough to promote phase separation. Curli are the major protein component of the biofilm extracellular matrix^61^. Biofilms are formed as a response to environmental stresses such as pH, temperature, and high salt, among others^62^. The ability of CsgF to phase-separate at a wide range of pH and salt concentration indicates that curli/biofilm could potentially form and withstand adverse conditions (Supplementary Fig. 1b). The presence of disordered N-terminus in CgsF-homologs also suggests that phase separation might be a common theme in many bacterial strains (Supplementary Fig. 5). It was previously demonstrated that *csgA*^*‒*^ cells accept CsgA from donor cells (*csgB*^*‒*^ cells) in a process called interbacterial complementation^12^. Therefore, we anticipated that *csgF*^*‒*^ cells might recruit CsgF to the cell surface if the protein is available outside the cell. Purified CsgF added exogenously complemented *csgF*^*‒*^ cells (Fig. 3c, d). CsgF-∆N or CsgF-∆C failed to complement *csgF*^*‒*^ cells (Fig. 3a–c). Thus, both the N- and C-termini are essential for the function of CsgF. Since the CsgF N-terminus is involved in CsgG interaction, it is likely that the C-terminus mediates interactions with CsgB. Immunofluorescence assays on curli-expressing cells (MC4100) showed that CsgF localized on the cell surface as puncta, similar to what was observed previously in BW25113 (Fig. 5f)^24^. We find here that the presence of surface-localized CsgF regulates the secretion of CsgA to the outer membrane (Fig. 5a–g).

**Fig. 7:**
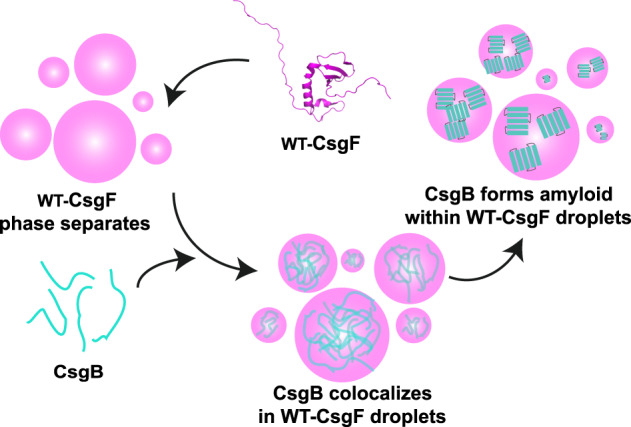
Model to show CsgB amyloid formation in the CsgF droplets. WT-CsgF (PDB ID: 5M1U) forms biomolecular condensates. SDS-soluble monomeric CsgB is recruited in the WT-CsgF droplets and undergoes amyloid formation.

CsgF is required for anchoring CsgB to the cell surface, without which cells fail to properly nucleate curli assembly^12,21^. CsgF induces the formation of protease-resistant CsgB on the cell surface^21^. We find here that the curli nucleator protein CsgB does not phase-separate on its own (Supplementary Fig. 5a). However, in the presence of CsgF, CsgB colocalized with CsgF condensates and transitioned from a dynamic state into a less dynamic amyloid state (Fig. 6). Scaffolds promote assembly and maintain the integrity of biomolecular condensates, whereas clients are biomolecules that are recruited into condensates, but are not necessary for their formation^37,63,64^. Clients with an affinity to scaffolds can be recruited into condensates^63^. CsgB is not required to promote the phase separation of CsgF but is localized within the CsgF droplets (Figs. 1d, e, and 6a). Thus, CsgB is a client for the CsgF scaffold. It was shown previously using a pull-down assay that CsgF interacts with CsgB but not with CsgA^23^. CsgA does not sequester to CsgF condensates like CsgB (Supplementary Fig. 5c). The CsgB∆R5 mutant (CsgB without C-terminus R5 repeat) of CsgB is also recruited to CsgF droplets (Supplementary Fig. 5b). In the absence of both the middle and C-terminal regions, CsgF does not interact with CsgB^23^. Ectopic expression of ∆C-CsgF in a *csgF*^*‒*^ mutant strain revealed that ∆C-CsgF is bound to the cells but does not complement *csgF*^*‒*^ cells to WT levels of curli production (Fig. 3a–d), suggesting that the C-terminal 11-amino acid residues are interacting with the N-terminus of CsgB on the bacterial outer membrane. CsgB can nucleate the aggregation of CsgA^8,9,55,65^. Proteins that are associated with neurodegenerative diseases, such as Tau, TDP-43, and FUS, have the propensity to undergo phase separation and to aggregate within the droplets^44,45,66^. Condensates can promote or inhibit the aggregative properties of any client protein^45,67,68^. Condensates of human prion protein (PrP) can recruit amyloid-β (Aβ) oligomers, and this results in the formation of a hydrogel with immobile Aβ and relatively mobile PrP^69^. A recent study has shown that Aβ–42 sequesters in other biomolecular condensates such as Laf1, Dbp1, and Ddx4, which leads to a lower aggregation propensity of Aβ–42^68^. CsgB recruited in the CsgF condensate became less dynamic with time and SDS-PAGE on CsgF-CsgB reaction mixture indicated a time-dependent formation of SDS-insoluble CsgB aggregates (Figs. 6 and 7). In the case of disease-causing amyloids, it has been shown that the intermediates can be toxic and disrupt the membrane, which ultimately leads to cell death^70^. Bacterial cells must make curli without inducing membrane toxicity. CsgF phase separation might be a way to sequester amyloid intermediates at a specific location to avoid toxicity. Functional amyloid-forming systems might have a different mechanism to impede the formation of toxic amyloid intermediates. Based on this evidence, we propose that concentrating CsgB into CsgF droplets triggers the formation of amyloid nuclei that promotes and spatially regulates CsgA aggregation specifically on the cell surface.

In this study, we demonstrate that the curli accessory protein CsgF undergoes phase separation. Phenylalanine residues in the N-terminal region are stickers responsible for the assembly of CsgF condensates. Without the N-terminal region, CsgF failed to secrete to the cell surface, and both the N-terminal and C-terminal regions are required for the proper function of CsgF on the outer membrane. Exogenously added CsgF protein complemented *csgF*^*‒*^ cells. The curli nucleator protein CsgB acted as a client for the CsgF scaffold. CsgF condensates provided a platform to promote the aggregation of CsgB (Fig. 7). CsgF assembled on the outer membrane governed the secretion of CsgA. Taken together, this study provides insights into the interplay of multicomponent CsgF condensate and its influence on curli amyloid biogenesis.

## Methods

### Protein purification

Amino acid sequences of all the proteins used in this study are given in Supplementary Table 1. WT-CsgF with C-terminus His-tag was expressed and purified as described previously with modifications^47^. CsgF pET11d in BL21 (DE3) and CsgF variants were overexpressed using 500 µM IPTG for 4 h at 37 °C. The cell pellet from 250 mL culture was lysed in 25 mL of 8 M GdmCl, 150 mM NaCl, and 50 mM potassium phosphate (KPi) pH 7.3, and the denatured solution was kept on a shaker overnight at room temperature (RT). The lysate was centrifuged, and the supernatant was sonicated three times with pulse-on for 30 s and pulse-off for 30 s. To the sonicated lysate, 2.5 mL of Ni-NTA beads were added and allowed to bind for an hour at RT. Ni-NTA beads were washed with four different solutions. Wash 1: 10 mL of 8 M urea, 650 mM NaCl, 50 mM KPi pH 7.3. Wash 2: 6 mL of 4 M urea, 50 mM KPi pH 7.3. Wash 3: 6 mL of 2 M urea, 20 mM imidazole, 50 mM KPi pH 7.3. Wash 4: 6 mL of 2 M urea, 40 mM imidazole,50 mM KPi pH 7.3. Then the protein was eluted in 2 M urea, 200 mM imidazole, and 50 mM KPi pH 7.3. The eluents were dialyzed against 1% acetic acid and lyophilized. The protein was stored at −20 °C for further use. The molar extinction coefficient used to estimate the concentration of WT-CsgF, CsgF-∆N, CsgF-∆C were 3840 M^−1^cm^−1^, 2560 M^−1^cm^−1^, and 3840 M^−1^cm^−1^ (Scripps calculator), respectively. For all phenylalanine mutants, the molar extinction coefficient used was 3840 M^−1^cm^−1^. The plasmids used in the study are listed in Supplementary Table 2. CsgF truncation and phenylalanine mutants were made using primers from Integrated DNA Technologies and the list of primers is provided in Supplementary Table 3. The PCR reactions were performed using Phusion High-Fidelity Master Mix (Thermo Scientific, catalog number: F531L).

CsgA, CsgB, and CsgB∆R5 were purified as described elsewhere^56,71^. Briefly, CsgA and CsgB∆R5 overexpressed cell pellet from 250 mL culture was lysed with 25 mL of 8 M GdmCl, 50 mM KPi pH 7.3. The lysate was centrifuged, and the supernatant was sonicated three times with pulse-on for 30 s and pulse-off for 30 s. The lysate was incubated with Ni-NTA beads for an hour. The Ni-NTA beads were washed first with 50 mM KPi pH 7.3 and then with 12.5 mM imidazole 50 mM KPi pH 7.3. The CsgA and CsgB∆R5 were eluted with 125 mM imidazole 50 mM KPi pH 7.3. For CsgB purification cell pellet (250 mL) was resuspended in 50 mM KPi pH 7.3 and sonicated eight times with pulse-on for 30 s and pulse-off for 30 s. To the pellet, 25 mL of 8 M GdmCl, 150 mM NaCl, 50 mM KPi pH 7.3. was added and allowed to denature overnight. The denatured solution was centrifuged, and to the supernatant, Ni-NTA beads were added and allowed to bind for an hour at RT. The Ni-NTA beads were washed first with 8 M urea, 650 mM NaCl, 50 mM KPi pH 7.3, and then with 8 M urea, 150 mM NaCl, 50 mM KPi pH 7.3. and the final wash was given with 8 M urea, 150 mM NaCl, 12.5 mM imidazole 50 mM KPi pH 7.3. CsgB was eluted with 8 M urea, 150 mM NaCl, 125 mM imidazole 50 mM KPi pH 7.3. The eluents were buffer exchanged with 25 mM potassium phosphate pH 7.5 (Zeba spin 5 mL column and the concentrations were estimated by BCA assay.

McdB purification was carried out as described previously^72^. McdB with N-terminus His-SUMO tag in pET11b vector was overexpressed in BL21-AI cells with 1 mM IPTG and 0.2% L-arabinose. The cell pellet from 1 L culture was resuspended in 30 mL of 300 mM KCl, 50 mM Tris-HCl pH 8.4, 5 mM β-mercaptoethanol (BME), 50 mg lysozyme, one protease inhibitor tablet. The cells were sonicated with cycles of 10-s pulse-on and 20-s pulse-off for 7 min. The lysate was centrifuged, the supernatant was passed through a 0.45-µm filter and the filtrate was loaded onto a 1 mL HisTrap column that was preequilibrated with 300 mM KCl, 50 mM Tris-HCl pH 8.4, 5 mM BME. The column was washed with 5 mL of 300 mM KCl, 25 mM imidazole, 50 mM Tris-HCl pH 8.4, and 5 mM BME. McdB was eluted using a 5–100% gradient of 300 mM KCl, 500 mM imidazole, 50 mM Tris-HCl pH 8.4, 5 mM BME. The His-SUMO tag was cleaved from McdB using Ulp1 protease (protease: Sample = 1:100). The samples were concentrated and loaded onto size exclusion chromatography column HiLoad 16/600 Superdex 200 pg that was preequilibrated with 150 mM KCl, 20 mM CAPS pH 10.2, 5 mM BME, 10% glycerol. The concentrated fractions were stored at −80 °C for further use. McdB concentration was estimated using the molar extinction coefficient of 18,115 M^−1^cm^−1^.

### Labeling of proteins

Proteins were either dissolved or buffer exchanged in 8 M urea, 100 mM sodium carbonate pH 8.3 buffer and kept at room temperature for an hour and a half with mild agitation. NHS-Alexa dyes or FITC (Invitrogen) to protein molar ratio used was 2:1. The reaction was carried out at room temperature for 1.5 h. Free dye from the CsgF sample was removed by dialysis against 25 mM Tris pH 9 buffer. Dialyzed CsgF protein was then passed through a 5-mL Zeba spin desalting column (Thermo Fisher) that was equilibrated with 25 mM Tris pH 9 buffer. For CsgA and CsgB, 1 mL reaction mixture was diluted in 15 mL 8 M urea, 50 mM KPi pH 7.5 buffer, and concentrated using a 3 kDa filter (Sartorius) to remove excess dye. The concentrated solution was buffer exchanged with 50 mM KPi pH 7.3 buffer using a 5 mL Zeba spin desalting column. The concentration of labeled proteins was estimated using the molar extinction coefficient ε_494_ = 73,000 M^−1^cm^−1^ (Alexa-488), ε_632_ = 159,000 M^−1^cm^−1^ (Alexa-633), ε_280_ = 10810 M^−1^cm^−1^ (CsgA), ε_280_ = 7680 M^−1^cm^−1^ (CsgB), and ε_280_ = 3840 M^−1^cm^−1^ (CsgB).

### Turbidity measurements

For this, 1.0 mM stocks of CsgF or CsgF variants were prepared in 1% acetic acid. The protein was diluted in 100 µL of 25 mM potassium phosphate pH 7.5 buffer, and absorbance at 350 nm was recorded immediately using a TECAN infinite 200 PRO plate reader with Tecan i-control software. The data for the turbidity measurements represent the mean with standard deviations (SD) obtained from three independent measurements. Source data are provided in the Source Data file.

### Imaging and FRAP measurements

Imaging was performed on a 16-well glass-bottom culture well (Grace BioLabs), which was passivated with 5% (W/V) pluronic acid (Sigma Aldrich) for 2 h and washed thrice with 25 mM potassium phosphate pH 7.5 buffer. Labeled: Unlabeled protein used was 1:50. Then, 1 mM CsgF was prepared in 1% acetic acid and diluted to the indicated buffer. Samples were imaged within 15–20 min either on a Leica DMI6000B inverted microscope (63× objective) or on a Nikon Ti2-E motorized inverted microscope (100× objective). For imaging CsgF together with CsgB, the required volume of CsgF was added from 100 µM CsgF, 25 mM potassium phosphate pH 7.5 (diluted from 1 mM CsgF, 1% acetic acid) to CsgB at room temperature. For the experiments where McdB was mixed with CsgB, 100 µM McdB droplets were prepared in 100 mM NaCl, 25 mM potassium phosphate pH 7.5 buffer, and then the required volume was added to CsgB. FRAP measurements were carried out using a Nikon Ti2-E motorized inverted microscope controlled by NIS Element software, a 100× objective (Oil CFI60 Plan Apochromat Lambda Series for DIC), a photometric Prime 95B Black-illuminated CMOS camera, and a SOLA 65 LED light source. Alexa-488 labeled CsgB was imaged using a GFP filter set [excitation, 470/40 nm (450–490 nm); emission, 525/50 nm (500–550 nm); dichroic mirror, 495 nm]. Alexa-633 labeled CsgF was imaged using a CY5 filter set [excitation, 620/60 nm (590–650 nm); emission, 700/75 nm (663–738 nm); dichroic mirror, 660 nm]. The region of interest was bleached with a 405-nm laser at 40% power (20 mW) with a 200-μs dwell time. Post-bleach images were acquired every 3 s for 5 min. The images were analyzed using Fiji/ImageJ software. The bleached region was background corrected and normalized with the prebleached fluorescence intensity. The data was exported and plotted using OriginPro 2019 software. The FRAP data represent the mean with a standard deviation from 10 or more bleached areas. The FRAP source data is provided in the Source Data file. Images in Leica DMI6000B inverted microscope collected with inbuilt LAS AF software.

### Sedimentation assay

In this, 20 µM of 100 µL protein solution was prepared in 25 mM potassium phosphate pH 7.5 and allowed to stand at room temperature for 20 min. The protein samples were centrifuged at 16,000×*g* for 2 min. To the pellet, 100 µL of 8 M urea, 50 mM potassium phosphate pH 7.5 was added and the samples were boiled and subjected to electrophoresis on a 15% acrylamide gel. SDS-PAGE gels were imaged using an Alpha Innotech FluorChem SP system using FluorChem SP (AIC) software. The Coomassie-stained gels were quantified using Fiji/ImageJ. The data was plotted in OriginPro 2019. The uncropped gel images are provided in the Source Data file. The gels shown for sedimentation assays are representative of three independent experiments with similar observations.

### Congo red assay

The bacterial cells that were grown overnight with 50 µg/mL kanamycin were normalized to 1 OD_600_ in LB media and 4 µL cells were spotted on YESCA (Yeast Extract Casamino Acid) agar plate containing 50 µg/mL Congo red dye. For the exogenous complementation assay, protein stocks (1, 10, 20, and 30 µM) were prepared in 25 mM Tris pH 9 buffer. Then, 4 µL of protein was spotted on the YESCA-Congo red plate and air-dried. On the protein spot, 4 µL of 1 OD_600_ cells were spotted, and the plate was incubated at 26 °C for 48 h. The Congo red plates were imaged using a Canon EOS Rebel XSi camera and the background was changed using Adobe Photoshop 2022. The bacterial strains used in this study are provided in Supplementary Table 4. The Congo red plates shown in the Figures are representative of three independent experiments with similar observations. The raw images are provided in the Source Data file.

### Western blots and dot blots

The whole-cell and plug assay were carried out as described elsewhere^71^. Briefly, the cells streaked on a YESCA agar plate and grown for 48 h at 26 °C were scraped off and resuspended in 50 mM potassium phosphate pH 7.3 buffer. Next, 150 µL of cells (1 OD_600_) were centrifuged for 10 min at 16,000×*g*. To the cell pellet, 100 µL of 88% formic acid was added and speed vacuumed to obtain formic acid-treated whole-cell samples. For the plug assay, the agar with cells was stabbed with the back side of a 1-mL pipette tip and carefully transferred to a 1.5-mL microcentrifuge tube using tweezers. Then, 100 µL of 88% formic acid was added to the plug and vortexed until most of the plug was dissolved. The formic acid was removed from the samples using a speed vacuum at 55 °C for 2 h. To the formic acid and non-treated samples, 50 µL of 2x SDS-PAGE (polyacrylamide gel electrophoresis) running buffer and 10 µL of SDS-PAGE loading buffer were added. The samples were boiled for 10 min and subjected to electrophoresis. To the agar plugs that were treated with formic acid, 1 µL 0.5 M NaOH was added before boiling the samples. The transfer was carried out in 25 mM CAPS pH 11 buffer at 11 V for 15 h. For CsgA antibody staining, a PVDF membrane was used, and a nitrocellulose membrane was used for probing CsgF. The dilutions used were 1:10,000 for CsgA antibody and 1:2500 for CsgF antibody. All the washes were performed in TBS-T buffer. For the intact cell dot blot, 4 µL of 1 OD_600_ cell were spotted on the nitrocellulose membrane, and blotting was performed in PBS buffer. IRDye 800CW goat anti-rabbit secondary antibody (Li-COR) at a 1:10,000 dilution was used, and the membranes were imaged using a Li-COR ODYSSEY with inbuilt Image Studio Ver 5.2 software. The bands were quantified using ImageJ. Statistical analysis was performed using one-way ANOVA with the Tukey test in OriginPro 2019. Uncropped and unprocessed blot images are provided in the Source Data file. CsgA and CsgF antibodies are made by Proteintech Group Inc upon request. We validated CsgA and CsgF antibodies by performing western blot on MC4100 bacterial wild-type cells and CsgA/ CsgF mutant cells.

### Immunofluorescence staining

Cells were adhered to a polylysine-treated glass slide and fixed with 4% formaldehyde. The glass slides were blocked with 5% BSA for an hour and then treated with anti-6x His-tag antibody (ABGENT, catalog number: AM1010A) at 1:100 dilution for an hour at room temperature. After washing three times with PBS, the cells were stained with the goat anti-Mouse IgG Alexa-568 labeled secondary antibody (Invitrogen, catalog number: A-11004) at 1:1000 dilution. The cells were washed thrice with PBS and stained with DAPI. Imaging was performed on a Leica SP8 inverted scanning confocal microscope (LAS X software) with a 100× oil immersion objective lens. Immunofluorescence staining was performed three times with similar observations.

### Bioinformatic and structural predictions tools

The prion-like amino acid composition [http://plaac.wi.mit.edu/] was used to analyze the prion nature of CsgF. The sequence alignment of CsgF-like proteins was performed using the Network Protein sequence analysis [https://npsa.lyon.inserm.fr/]. The structure of CsgF-like proteins was predicted using AlphaFold Protein Structure Database [https://alphafold.ebi.ac.uk/].

### Reporting summary

Further information on research design is available in the Nature Portfolio Reporting Summary linked to this article.

## Supplementary information

Supplementary Information

Description of Additional Supplementary Files

Supplementary Movie 1

Supplementary Movie 2

Reporting Summary

## Data Availability

The data are available within the Article, Supplementary Information, or Source data file. PDB (Protein Data Bank) ID used in this study is available on the PDB web server via the accession code 5M1U. The protein sequences used for alignment are available in UniProt [https://www.uniprot.org/]. Source data are provided with this paper.

## Source data

Source Data
