# Supplementary material for: Cell surface-localized CsgF condensate is a gatekeeper in bacterial curli subunit secretion
Source: Nat Commun. 2023 Apr 26;14:2392. doi: 10.1038/s41467-023-38089-1 (PMC10133297; doi:10.1038/s41467-023-38089-1)
Supplement: Supplementary file 1 — Supplementary Information [file 41467_2023_38089_MOESM1_ESM.pdf]

Supplementary Information

Cell Surface-localized CsgF Condensate is a Gatekeeper in Bacterial Curli Subunit Secretion

Hema M. Swasthi, Joseph L. Basalla, Claire E. Dudley, Anthony G. Vecchiarelli, and Matthew R. Chapman*

Department of Molecular, Cellular, and Developmental Biology, University of Michigan, Ann Arbor, MI 48109-1048 USA

*Correspondence: chapmanm@umich.edu

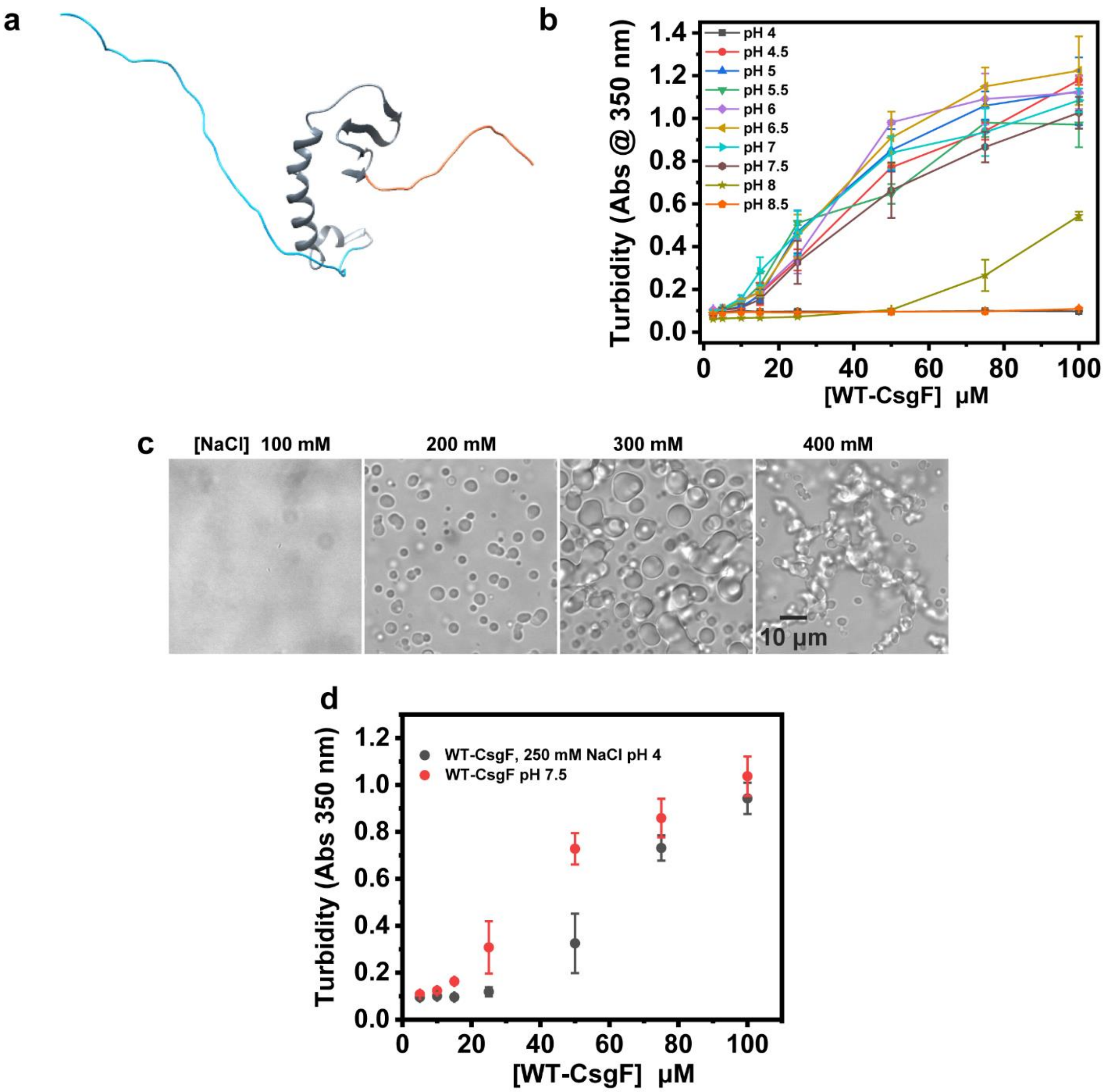

Supplementary Fig. 1: (a) The NMR structure of WT-CsgF (PDB ID: 5M1U) [https://www.wwpdb.org/pdb?id=pdb_00005m1u]. **(b)** Turbidity measurements of WT-CsgF from pH 4 to 8.5. The data represent mean ± SD, n=3 **(c)** DIC images of 100 µM WT-CsgF at pH 4 in the presence of varying concentrations of NaCl. The imaging was performed three times with similar observations. **(d)** Turbidity at varying concentrations of WT-CsgF in 50 mM potassium phosphate pH 7.5 and in the presence of 250 mM NaCl, 25 mM sodium citrate pH 4. The data represent mean ± SD, n=3.

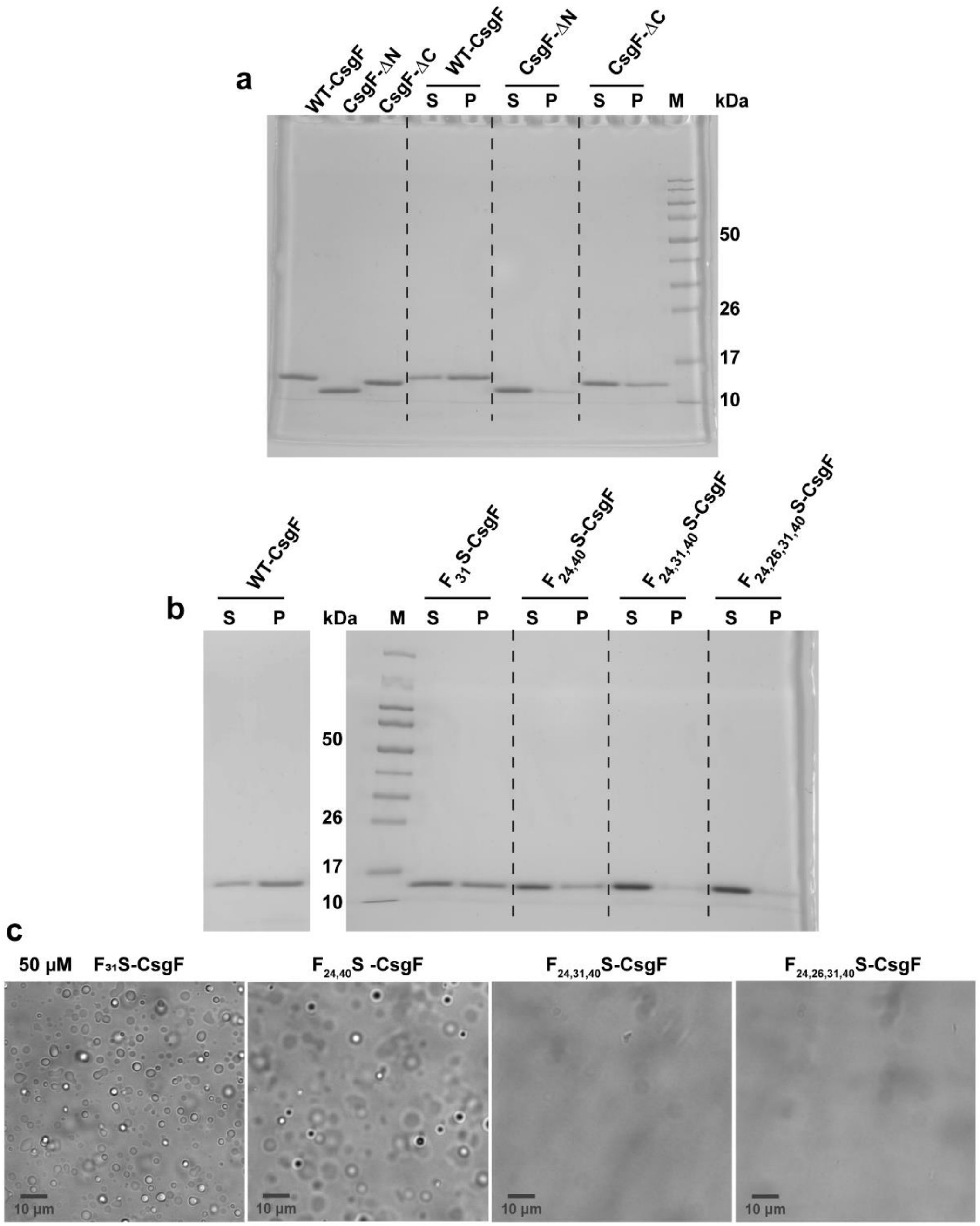

Supplementary Fig. 2: (a) Sedimentation assays performed on 20 μM WT-CsgF, CsgF-ΔN, and CsgF-ΔC were run on SDS-PAGE and stained with Coomassie blue. M: Molecular weight marker, S: Supernatant, P: Pellet **(b)** Coomassie-stained SDS-PAGE on the supernatant and pellet samples of 20 μM CsgF phenylalanine mutants after sedimentation. WT-CsgF was run on a separate gel from the phenylalanine mutant samples but the gels were run and processed in parallel. M: Molecular weight marker, S: Supernatant, P: Pellet. For sedimentation assays, 100 μL samples were incubated for 20 min at room temperature before centrifugation. **(c)** DIC images of 50 μM phenylalanine CsgF mutants in 50 mM potassium phosphate pH 7.5 buffer. The experiments were performed three times with similar observations (**a, b, c**).

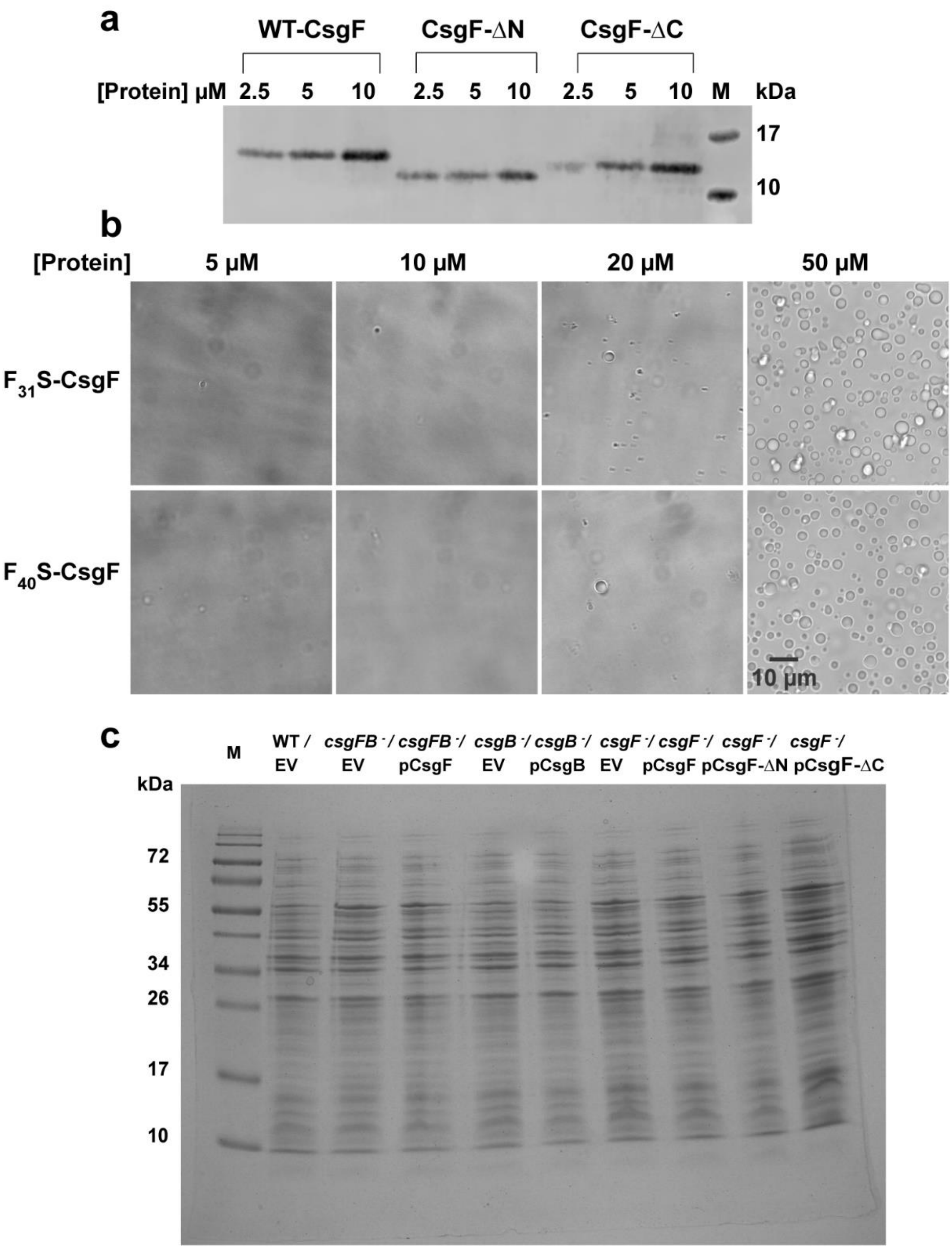

Supplementary Fig. 3: (a) Western blot on purified WT-CsgF, CsgF-ΔN, and CsgF-ΔC probed with CsgF antibody on PVDF membrane. M: Molecular weight marker **(b)** DIC images of 5 μM, 10 μM, 20 μM, and 50 μM of F_{31}S-CsgF and F_{40}S-CsgF. **(c)** Coomassie-stained SDS-PAGE gel for the samples shown in Figure 5d. M: Molecular weight marker, EV: Empty vector. The experiments were performed in triplicate with similar observations (**a, b, c**).

a

N-terminus

```
Q1RDC3_ECOUT       ..GTMTFQFR NPNFGG.NPN NGAFLLNSAQ AQNSYKDPSY ......NDDFG....IETPS ALDNFTQAIQ SQILGGLLSN INTGKPGRMV TN........
Q88H88_PSEPK       ..TELIYEPV NPNFGG.NPL NGSYLLNNAQ AQDRHKDPDQ ......LDSL.....YEQPS ALDRFTDSLE TRLLNQLLTD VGNGNSGELI TD........
A0A143HJA0_9GAMM   ..SELVYTPV NPNFGG.NPL NTSHLFGGAN AINDYKGP.Q ......EDSL.....FEQES ALDRLTSSLQ SRLISQLLAD VGNGNTGQLI TD........
Q5E245_ALIF1       ..TQLVYTPV NPAFGG.SYL NGSYLLANAS AQNEHQ.... ......GGSS.....YVAPT ALDRLASSLQ SRLMSQLFND AANGGEGYLK TD........
Q084E5_SHEFN       ..TQLIYTPV NPNFGG.SYL NGSYLLANAS VQNDHK.... ......TSSG.....YTPPT ALERMANSLE SRLMSQLFND AANGGEGYLK TT........
D4ZLW2_SHEVD       ..TELVYTPK NPAFGG.SPL NGSYLLGNAQ AQNDYDDP.. ......NAKK.....ATTLS ALERFTNQLQ SSLLSDVLAK ARKGEAGSLQ TD........
N2IYT1_9PSED       .SSSLVYEPV NPTFGG.NPL NTTHLFSRAE AINDYEGS.S ......GSS.......SSSS VLSSLTSSLQ SRLISQLLA. ..TGEAGSLE TN........
F0LZU2_VIBFN       ..GQLIYQPI NPSFGG.DPL LGNHLLNKAQ AQDTKRDPNA ......PDFG.......RFS ETEFFIQDLR AKLINDAIQD AVDGGDGESI ID........
W7QHV5_9GAMM       ..TELVYEPI NPSFGG.NPL NGSFLLSKAN SQNAHTAPYS .................ERS YDERLQESLE RAYINRIVRE ITDIAFGEQE YDEDGNPIDS
A0A136HQR0_9ALTE   ..TEIVYQPI NPSFGG.NPM NGSFLLQKAQ SQNAHTAPDE .................GRS FVDRFRDALE RNIINSLTRR IADGEIVEGL YD........
A0A0W1SRL3_9GAMM   ..TELVFSFT NPSFGG.DPM IGNFLLNKAD SQKRFAEDEY .......E..........ED PLSDFEDGLN RRVLGLIADK IVQDAFGDEG NLSN......
F3Z094_DESAF       ..SSLVYQPV NPAFGG.PQL NGSWLQAEAN AQNIPQAAAQ RRQ.QITGAAASAARGGILT PGQAFAQQLQ TQLYSSLANQ ITRAIFGENA QP........
B0UH01_METS4       DAGSLVYTPT NPAFGG.SPL NGSWQMQQAT AGNHFNRAAP .....TSGPQQ.......LT QSQIFAQQLQ SQLYASLANQ VTQAIFGANA QQ........
Q6NAU5_RHOPA       ..QQLIYQPT NPSFGG.YAA NTTHLFATAN AQKTATVGGS NKYGSGSGSGADGSGS...S LADLFVRQLQ NRLIYALADQ VSQAIFGDDP KD........
G8PUY5_PSEUV       ..GDLVYTPV NPSFGG.SPL NSAHLLSIAG AQKNATASDY KKPVDSTSAGTTGSGTKTQS DADLFVRQLQ GRLLSALASQ VTDAIFGENP QD........
A0A0S2ETP7_9HYPH   ..AELGYTPV NPSFGG.SPL NGSTLLSEAS AQ.KPNAPTS .....KTASST.......TS ASQQFLQMLQ SQLYASLASS VAQAVTGATT G.........
E3I1Z1_RHOVT       ..QQLVYKSI NPFFGGGDSF AYQQLLASAN AQNDFVE... .........ENSSLFSQSS. EIENFTDNLN RQLLNSLSQG LFQEQFG.SQ GLSEG.....
A0A176T7M2_9FLAO   ..QGLIYKPK NPAFGG.DTF NYQWLASSAE SQNKFTD... .........KTISQVEQT.. ELERFTSSLN SLLLSQISSS LFRQQFG.SD GIKIG.....
A0A167UJA2_9FLAO   ..QALVYHPN NPAFGG.NTF NYQWMLSSAQ AQDRLTDPTL ........KKTTATATKTSN QLSDLATSIQ RQLLSRITND LLKNQFGENS SLKEG.....
```

```
Q1RDC3_ECOUT       .......... .DYIVDIANR D.GQLQLNVT DRKTGQTSTI QVSGLQNNSTDF........ .......... .......... .....
Q88H88_PSEPK       .......... .DFIVQIVDN D.GVLTVLVT DRSTGDKSEI IVSGLNPTN........... .......... .......... .....
A0A143HJA0_9GAMM   .......... .DFILNIVDD S.GALLIQIV DKATGETSEI QVSGLTPD............ .......... .......... .....
Q5E245_ALIF1       .......... .DFEINVVNE D.GSLLVHIT DLLTGETTII EVGGIVGTTTGG........ .......... .......... .....
Q084E5_SHEFN       .......... .DFEINVVNE D.GTLLVHIT DIVTGETTVI EVGGLVNTGTGG........ .......... .......... .....
D4ZLW2_SHEVD       .......... .DFIVNMLKD ETGQLVIHIT DRATGEISEI VTG....TTY.......... .......... .......... .....
N2IYT1_9PSED       .......... .DYSLVITDN D.GYLTITIT DKATGESTDI QVSGL............... .......... .......... .....
F0LZU2_VIBFN       .........S STLRVELRSS GGGSFSMTIL DKTTGETTTV NFGQPF.............. .......... .......... .....
W7QHV5_9GAMM       IFNQDSIFVS GDFQVELITS NPDSIVVNIT NLLTGEVTVV EIPRFG.............. .......... .......... .....
A0A136HQR0_9ALTE   .........T GEYTVEVIGQ PDGSVLVYIT HNETGEQTVI TMPAI............... .......... .......... .....
A0A0W1SRL3_9GAMM   .....GTYTI GSYVISIDNN G.KVIKVAVT DSLSGDSTVV EVPVF............... .......... .......... .....
F3Z094_DESAF       ....NGQFSF QGTTISYTRV G.SNVSITIN DGST..VTTV TVPATP.............. .......... .......... .....
B0UH01_METS4       ....SGTFSF QGTTISFAKV D.GQTNITIN DGST..VTQI SLPTVTH............. .......... .......... .....
Q6NAU5_RHOPA       ....SGTVSF GDQKVSFVRG S.DSIQLQII DESTGAITDI TVPVLQTDTPIN........ .......... .......... .....
G8PUY5_PSEUV       ....SGTVQF GDTKVTFDRT L.TTIRLRIE NATDGTVTDI VVPQLVTSKPATTTASTASS SASASQLSTA TPVLSGSALT PLSIN
A0A0S2ETP7_9HYPH   .....GTIVL DNLTVTWAIA S.TGKVITMT DASTGQVTKI TVPIVTTSN........... .......... .......... .....
E3I1Z1_RHOVT       .....TY.VY GSLVVEVNPG V.GGLSVSIL NTSTGEQTQI TIPNN............... .......... .......... .....
A0A176T7M2_9FLAO   .....SY.TF GSLSVEVYPS S.GGLTVDIL DTKTGETTQV IIPNR............... .......... .......... .....
A0A167UJA2_9FLAO   .....TY.KY GDFTVTIRNQ Y.DGIYIRIV DNQGGE.TSI QVPYF............... .......... .......... .....
```

b

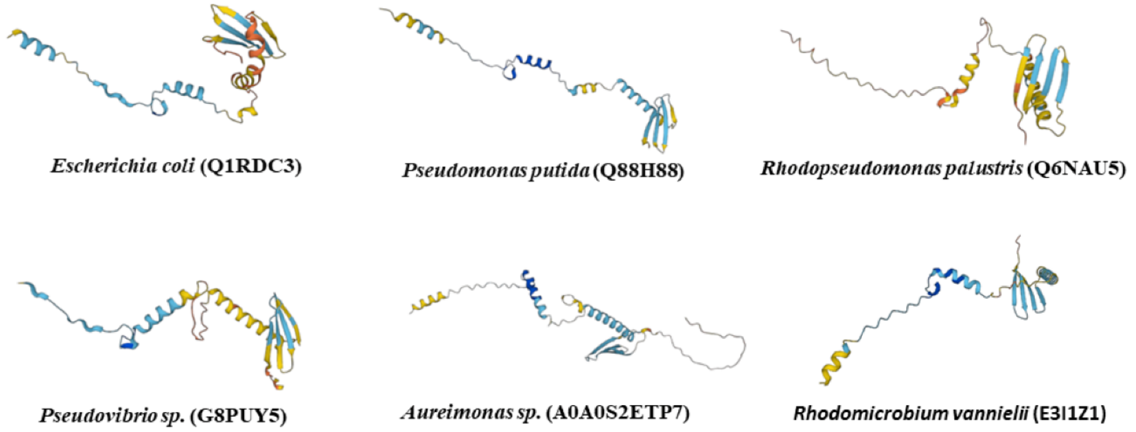

Supplementary Fig. 4: (a) Sequence alignment of CsgF homologs (UniProt IDs are given at the beginning of every sequence). Sequence alignment was performed using Network Protein Sequence Analysis software. Tyrosine and Phenylalanine residues in the N-terminal regions are highlighted in red. **(b)** AlphaFold predicted structures of CsgF homologs.

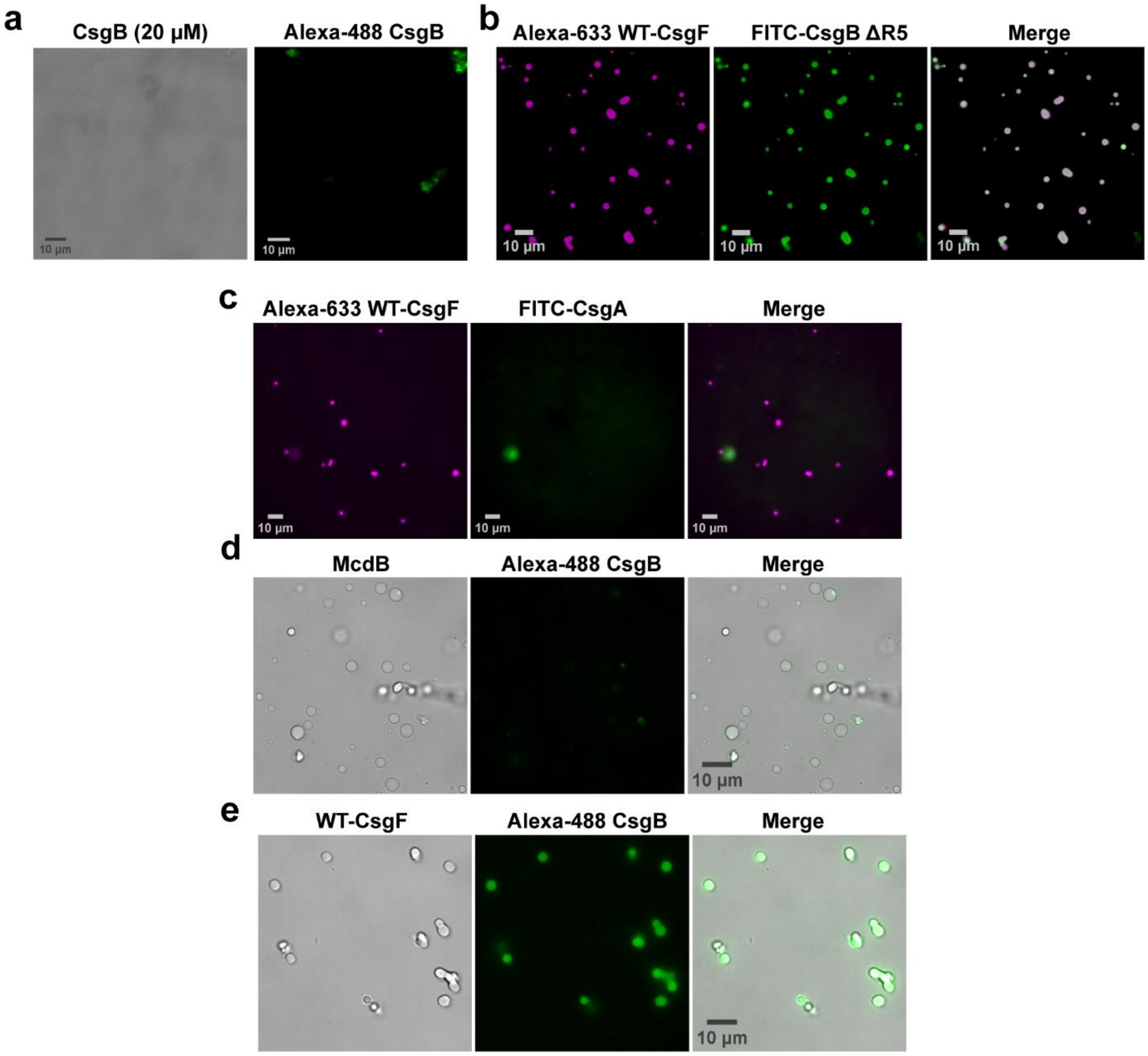

Supplementary Fig. 5: (a) DIC and fluorescence images of CsgB (20 µM) with Alexa-488 labeled CsgB. **(b)** Fluorescence images of Alexa-633 labeled WT-CsgF (20 µM) and 20 µM FITC-labeled CsgBΔR5 (CsgB consists of 5 imperfect repeats and CsgB without the C-terminus R5 repeat region is named as CsgB ΔR5). **(c)** Fluorescence images of Alexa-633 labeled WT-CsgF (20 µM) with FITC-labeled CsgA (20 µM). The experiments were carried out in 25 mM potassium phosphate pH 7.5 buffer. **(d)** Images of McdB (40 µM) (Maintenance of Carboxysome Distribution) in the presence of Alexa-488 labeled CsgB (20 µM). **(e)** Images of WT-CsgF (20 µM) with Alexa-488 labeled CsgB (20 µM). The experiments were carried out in 100 mM NaCl, 25 mM potassium phosphate pH 7.5 buffer. The molar ratio of unlabeled to the labeled protein used for fluorescence imaging was 50:1. The imaging was performed three times with similar observations (**a-e**).

Supplementary Table 1

Amino acid sequences of proteins used in the study

Protein	Sequence Information	Notes
WT-CsgF	MAGTMTFQFRNPNFGGNPNNGAFLLNSAQAQNSYKDPSY NDDFGIETPSALDNFTQAIQSQILGGLLSNINTGKPGRM VTNDYIVDIANRDGQLQLNVTDRKTGQTSTIQVSGLQNN STDFHHHHHH	Wildtype His-tagged CsgF
CsgF-ΔN	MASYNDDFGIETPSALDNFTQAIQSQILGGLLSNINTGK PGRMVTNDYIVDIANRDGQLQLNVTDRKTGQTSTIQVSG LQNNSTDFHHHHHH	Residues 20-54 from CsgF were deleted
CsgF-ΔC	MAGTMTFQFRNPNFGGNPNNGAFLLNSAQAQNSYKDPSY NDDFGIETPSALDNFTQAIQQILGGLLSNINTGKPGRMV TNDYIVDIANRDGQLQLNVTDRKTGQTSTIQHHHHHH	Residues 128-138 from CsgF were deleted
F_{24}S-CsgF	MAGTMTSQFRNPNFGGNPNNGAFLLNSAQAQNSYKDPSY NDDFGIETPSALDNFTQAIQSQILGGLLSNINTGKPGRM VTNDYIVDIANRDGQLQLNVTDRKTGQTSTIQVSGLQNN STDFHHHHHH	CsgF with F24S mutation
F_{26}S-CsgF	MAGTMTFQFRNPNFGGNPNNGAFLLNSAQAQNSYKDPSY NDDFGIETPSALDNFTQAIQSQILGGLLSNINTGKPGRM VTNDYIVDIANRDGQLQLNVTDRKTGQTSTIQVSGLQNN STDFHHHHHH	CsgF with F26S mutation
F_{31}S-CsgF	MAGTMTFQFRNPNSGGNPNNGAFLLNSAQAQNSYKDPSY NDDFGIETPSALDNFTQAIQSQILGGLLSNINTGKPGRM VTNDYIVDIANRDGQLQLNVTDRKTGQTSTIQVSGLQNN STDFHHHHHH	CsgF with F31S mutation
F_{40}S-CsgF	MAGTMTFQFRNPNFGGNPNNGASLLNSAQAQNSYKDPSY NDDFGIETPSALDNFTQAIQSQILGGLLSNINTGKPGRM VTNDYIVDIANRDGQLQLNVTDRKTGQTSTIQVSGLQNN STDFHHHHHH	CsgF with F40S mutation
$F_{24,40}$S-CsgF	MAGTMTSQFRNPNFGGNPNNGASLLNSAQAQNSYKDPSY NDDFGIETPSALDNFTQAIQSQILGGLLSNINTGKPGRM VTNDYIVDIANRDGQLQLNVTDRKTGQTSTIQVSGLQNN STDFHHHHHH	CsgF with F24S and F40S mutations
$F_{24,26,31,40}$S-CsgF	MAGTMTSQSRNPNSGGNPNNGASLLNSAQAQNSYKDPSY NDDFGIETPSALDNFTQAIQSQILGGLLSNINTGKPGRM VTNDYIVDIANRDGQLQLNVTDRKTGQTSTIQVSGLQNN STDFHHHHHH	CsgF with F24S, F26S, F31S and F40S mutations

CsgB	MAGYDLANSEYNFAVNELSKSSFNQAAIIGQAGTNNSAQ<br>LRQGGSKLLAVVAQEGSSNRAKIDQTGDYNLAYIDQAGS<br>ANDASISQGAYGNTAMIIQKGSGNKANITQYGTQKTAIV<br>VQRQSQMAIRVTQRHHHHHH	Wildtype His-tagged CsgB
CsgB-ΔR5	AGYDLANSEYNFAVNELSKSSFNQAAIIGQAGTNNSAQL<br>RQGGSKLLAVVAQEGSSNRAKIDQTGDYNLAYIDQAGSA<br>NDASISQGAYGNTAMIIQKGSGNKANITQYGTQKTAIVV<br>HHHHHH	19 residues from the C-terminal from CsgB were deleted
CsgA	MAGVVPQYGGGGNHGGGGNNSGPNSELNIYQYGGGNSAL<br>ALQTDARNSDLTITQHGGGNGADVGQGSDDSSIDLTQRG<br>FGNSATLDQWNGKNSEMTVKQFGGGNGAAVDQTASNSSV<br>NVTQVGFGNNATAHQYHHHHHH	Wildtype His-tagged CsgA
McdB	MTDAFDRLKKRSRTPIAREGSLTTGPELSDRPLQLLPRE<br>FETFCDRYAVHAGDVIEAALDLVLLDPDLQQRLLQRLRQ<br>GNGSDRVWLGTACPRSWQQQLQQQAQDQGLSEADLLQEA<br>IAQRLDLVLGQTTLREEVTLLRQELDQLKRKLHGW	Wildtype McdB

Supplementary Table 2

Plasmids used in the study

Plasmids	Relevant characteristics	References
pET11d	IPTG-inducible expression vector	New England Biolabs
WT-CsgF pET11d	C-terminal His_6 tagged *E.coli* CsgF cloned between Nco1/BamH1 sites of pET11d	[4]
CsgF-ΔN pET11d	C-terminal His_6 tagged without 20 to 53 residues of CsgF cloned between Nco1/BamH1 sites of pET11d	This study
CsgF-ΔC pET11d	C-terminal His_6 tagged without 127 to 138 residues of CsgF cloned between Nco1/BamH1 site of pET11d	This study
$F_{31}S$ CsgF pET11d	C-terminal His_6 tagged F31S CsgF cloned between Nco1/BamH1 site of pET11d	This study
$F_{24,40}S$- CsgF pET11d	C-terminal His_6 tagged F24S and F40S CsgF cloned between Nco1/BamH1 site of pET11d	This study
$F_{24,31,40}S$-CsgF pET11d	C-terminal His_6 tagged F24S, F31S, and F40S CsgF cloned between Nco1/BamH1 site of pET11d	This study
$F_{24,26,31,40}S$-CsgF pET11d	C-terminal His_6 tagged F24S, F26S, F31S, and F40S CsgF cloned between Nco1/BamH1 site of pET11d	This study
CsgA pET11d	C-terminal His_6 tagged *E.coli* CsgA cloned between Nco1/BamH1 site of pET11d	[5]

CsgB pET11d	C-terminal His_6 tagged *E.coli* CsgB cloned between Nco1/BamH1 site of pET11d	[6]
CsgB ΔR5	C-terminal His_6 tagged *E.coli* CsgB without R5 repeat was cloned between Nco1/BamH1 site of pET11d	[7]
pLR1	*csgBA* promoter in pACYC177 vector	[8]
CsgF pLR1	*E.coli* CsgF is cloned between Nco1/Pst1 of pLR1	[9]
CsgF-ΔN pLR1	*E.coli* CsgF without 20 to 53 residues cloned between Nco1/Pst1 of pLR1	This study
CsgF-ΔC pLR1	*E.coli* CsgF without 127 to 138 residues cloned between Nco1/Pst1 sites of pLR1	This study
CsgB pLR2	*E.coli* CsgF is cloned between Nco1/BamH1 of pLR2	[7]
CsgF-His pTrc99A	E.coli Csg-His cloned between Ecor1 and BamH1 sites of pTrc99A	[7]

Supplementary Table 3

Primers used in the study

Primers	Primer sequence
His CsgF-ΔN F<br>His CsgF-ΔN R	5’ CATGCCATGGCAAGCTATAACGATGAC 3’<br>5’ GCCGGATCCTTAGTGATGGTGATG 3’
His CsgF-ΔC F<br>His CsgF-ΔC R	5’ TCGACCATCCAGCATCACCATCACCATC 3’<br>5’ TGATGGTGATGCTGGATGGTCGAGGTTTG 3’
CsgF F24S F<br>CsgF F24S R	5’ GAACCATGACTAGCCAGTTCCGTAATC 3’<br>5’ GATTACGGAACTGGCTAGTCATGGTTC 3’
CsgF F26S F<br>csgF F26S R	5’ GACTTTCCAGAGCCGTAATCCAAAC 3’<br>5’ GTTTGGATTACGGCTCTGGAAAGTC 3’
CsgF F31S F<br>CsgF F31S R	5’ GTAATCCAAACAGTGGTGGTAAC 3’<br>5’ GTTACCACCACTGTTTGGATTAC 3’
CsgF F40S F<br>CsgF F40S R	5’ CCAAATAATGGCGCTAGTTTATTAAATAGC 3’<br>5’ GCTATTTAATAAACTAGCGCCATTATTTGG 3’
Sec-CsgF-ΔN F<br>Sec-CsgF-ΔN R	5’ GTTGGGCTAGCTATAACGATGACTTTGG 3’<br>CATCGTTATAGCTAGCCCAACTTAATGG
Sec-CsgF-ΔC F<br>Sec-CsgF-ΔC R	5’ TATTGACGACGGGATCAGTACC 3’<br>5’ CAAACTGCAGTTACTGGATGGTCGAGGTTTGTCC 3’

Supplementary Table 4

Strains used in the study

Common Name	Strains	Relevant Genotype	References
Wild-type	MC4100	F- *araD*139 Δ(*argF-lac*) U169 *rpsL*150(*strR*) *relA*1 *fibB*5301 *deoC*1 *ptsF*25 *rbsB*	1
csgF −	MHR592	MC4100 Δ*csgF*	2
csgFB −	MHR422	MC4100 Δ*csgF csgB*	2
csgB −	MHR261	MC4100 Δ*csgB*	3
BL21(DE3)		F-, ompT, hsdSB(rB-, mB-), dcm, gal, λ(DE3)	New England Biolabs
NEB3016		MiniF *lacIq*(CamR) */ fhuA2 lacZ::T7 gene1 [lon] ompT gal sulA11 R(mcr-73::miniTn10*--TetS*)2 [dcm] R(zgb-210::Tn10*--TetS*) endA1 Δ(mcrC-mrr)114::IS10*	New England Biolabs

Supplementary References

1. Casadaban, M. J. Transposition and fusion of the lac genes to selected promoters in Escherichia coli using bacteriophage lambda and Mu. *J. Mol. Biol.* **104**, 541–555 (1976).

2. Chapman, M. R. *et al.* Role of Escherichia coli Curli Operons in Directing Amyloid Fiber Formation. *Science (80-.).* **295**, 851–855 (2002).

3. Hammar, M., Arnqvist, A., Bian, Z., Olsén, A. & Normark, S. Expression of two csg operons is required for production of fibronectin- and Congo red-binding curli polymers in Escherichia coli K-12. *Mol. Microbiol.* **18**, 661–670 (1995).

4. Schubeis, T. *et al.* Structural and functional characterization of the Curli adaptor protein CsgF. *FEBS Lett.* **592**, 1020–1029 (2018).

5. Cegelski, L. *et al.* Small-molecule inhibitors target Escherichia coli amyloid biogenesis and biofilm formation. *Nat. Chem. Biol.* **5**, 913–919 (2009).

6. Qin, S. *et al.* The E. coli CsgB nucleator of curli assembles to β-sheet oligomers that alter the CsgA fibrillization mechanism. *Proc. Natl. Acad. Sci.* **109**, 6502–6507 (2012).

7. Hammer, N. D., Schmidt, J. C. & Chapman, M. R. The curli nucleator protein, CsgB, contains an amyloidogenic domain that directs CsgA polymerization. *Proc. Natl. Acad. Sci.* **104**, 12494 LP – 12499 (2007).

8. Robinson, L. S., Ashman, E. M., Hultgren, S. J. & Chapman, M. R. Secretion of curli fibre subunits is mediated by the outer membrane-localized CsgG protein. *Mol. Microbiol.* **59**, 870–881 (2006).

9. Nenninger A. A, Robinson L. S., & Hultgren, S. J. Localized and efficient curli nucleation requires the chaperone-like amyloid assembly protein CsgF. *Proc. Natl. Acad. Sci.* **106**, 900–905 (2009).
