# Supplementary material for: Cell surface-localized CsgF condensate is a gatekeeper in bacterial curli subunit secretion
Source: Nat Commun. 2023 Apr 26;14:2392. doi: 10.1038/s41467-023-38089-1 (PMC10133297; doi:10.1038/s41467-023-38089-1)
Supplement: Supplementary file 2 — Description of Additional Supplementary Files [file 41467_2023_38089_MOESM2_ESM.pdf]

Description of Additional Supplementary Files

Supplementary Movie 1: Fusion of 50 µM CsgF phaseseparated droplets in 25 mM potassium phosphate pH 7.5 buffer.

Supplementary Movie 2: Fusion of 50 µM CsgF-ΔC phase-separated droplets in 25 mM potassium phosphate pH 7.5 buffer.
