# Supplementary material for: Cell surface-localized CsgF condensate is a gatekeeper in bacterial curli subunit secretion
Source: Nat Commun. 2023 Apr 26;14:2392. doi: 10.1038/s41467-023-38089-1 (PMC10133297; doi:10.1038/s41467-023-38089-1)
Supplement: Supplementary file 5 — Reporting Summary [file 41467_2023_38089_MOESM5_ESM.pdf]

nature portfolio

Corresponding author(s): Matthew R. Chapman

Last updated by author(s): Apr 12, 2023

Reporting Summary

Nature Portfolio wishes to improve the reproducibility of the work that we publish. This form provides structure for consistency and transparency in reporting. For further information on Nature Portfolio policies, see our Editorial Policies and the Editorial Policy Checklist.

Statistics

For all statistical analyses, confirm that the following items are present in the figure legend, table legend, main text, or Methods section.

n/a	Confirmed	
☐	☒	The exact sample size (*n*) for each experimental group/condition, given as a discrete number and unit of measurement
☐	☒	A statement on whether measurements were taken from distinct samples or whether the same sample was measured repeatedly
☐	☒	The statistical test(s) used AND whether they are one- or two-sided<br>*Only common tests should be described solely by name; describe more complex techniques in the Methods section.*
☒	☐	A description of all covariates tested
☒	☐	A description of any assumptions or corrections, such as tests of normality and adjustment for multiple comparisons
☐	☒	A full description of the statistical parameters including central tendency (e.g. means) or other basic estimates (e.g. regression coefficient) AND variation (e.g. standard deviation) or associated estimates of uncertainty (e.g. confidence intervals)
☐	☒	For null hypothesis testing, the test statistic (e.g. *F*, *t*, *r*) with confidence intervals, effect sizes, degrees of freedom and *P* value noted<br>*Give P values as exact values whenever suitable.*
☒	☐	For Bayesian analysis, information on the choice of priors and Markov chain Monte Carlo settings
☒	☐	For hierarchical and complex designs, identification of the appropriate level for tests and full reporting of outcomes
☒	☐	Estimates of effect sizes (e.g. Cohen's *d*, Pearson's *r*), indicating how they were calculated

Our web collection on statistics for biologists contains articles on many of the points above.

Software and code

Policy information about availability of computer code

Data collection	Turbidity measurements: TECAN infinite M200 Pro: Tecan i-control software<br>Fluorescence imaging: Leica DMI6000B inverted microscope: LAS AF software<br>FRAP measurements: Nikon Ti2-E motorized inverted microscope: NIS Element software<br>Immunofluorescence imaging: Leica SP8 inverted scanning confocal microscope: LAS X software<br>Western blot imaging: Li-COR ODYSSEY: Image Studio Ver 5.2 software<br>SDS-PAGE gel imaging: Alpha Innotech FluorChem SP: FluorChem SP (AIC) software<br>Protein structure prediction: AlphaFold Protein Structure Database
Data analysis	OriginPro 2019, Fiji/mageJ, Network Protein Sequence Analysis software, PyMOL 2.5.2, Prion-Like Amino Acid Composition

For manuscripts utilizing custom algorithms or software that are central to the research but not yet described in published literature, software must be made available to editors and reviewers. We strongly encourage code deposition in a community repository (e.g. GitHub). See the Nature Portfolio guidelines for submitting code & software for further information.

Data

Policy information about availability of data

All manuscripts must include a data availability statement. This statement should provide the following information, where applicable:

- Accession codes, unique identifiers, or web links for publicly available datasets
- A description of any restrictions on data availability
- For clinical datasets or third party data, please ensure that the statement adheres to our policy

All the data are available in the article or supplementary information. Source data are provided with this paper. PDB (Protein Data Bank) ID used in this study is available on the PDB web server. PDB ID: 5M1U [https://www.wwpdb.org/pdb?id=pdb_00005m1u]. The protein sequences used for alignment are available in UniProt [https://www.uniprot.org/].

Human research participants

Policy information about studies involving human research participants and Sex and Gender in Research.

Reporting on sex and gender	Not applicable for our study.
Population characteristics	Not applicable for our study.
Recruitment	Not applicable for our study.
Ethics oversight	Not applicable for our study.

Note that full information on the approval of the study protocol must also be provided in the manuscript.

Field-specific reporting

Please select the one below that is the best fit for your research. If you are not sure, read the appropriate sections before making your selection.

☒ Life sciences ☐ Behavioural & social sciences ☐ Ecological, evolutionary & environmental sciences

For a reference copy of the document with all sections, see nature.com/documents/nr-reporting-summary-flat.pdf

Life sciences study design

All studies must disclose on these points even when the disclosure is negative.

Sample size	Sample size was determined based on common practice in the field and the data reproducibility to obtain statistics. The sample size (n) is provided in the figure legends.
Data exclusions	No data were excluded during analysis.
Replication	All the measurements were performed at least three times. The precise data points are included in the figures.
Randomization	No human or animals subjects were not involved in this study so randomization is irrelevant.
Blinding	No human or animals subjects were not involved in this study so blinding is irrelevant.

Reporting for specific materials, systems and methods

We require information from authors about some types of materials, experimental systems and methods used in many studies. Here, indicate whether each material, system or method listed is relevant to your study. If you are not sure if a list item applies to your research, read the appropriate section before selecting a response.

Materials & experimental systems

n/a	Involved in the study
☐	☒ Antibodies
☒	☐ Eukaryotic cell lines
☒	☐ Palaeontology and archaeology
☒	☐ Animals and other organisms
☒	☐ Clinical data
☒	☐ Dual use research of concern

Methods

n/a	Involved in the study
☒	☐ ChIP-seq
☒	☐ Flow cytometry
☒	☐ MRI-based neuroimaging

Antibodies

Antibodies used

CsgA, CsgF, His-tag, goat anti-rabbit, and goat anti-mouse antibodies. Rabbit CsgA antibodies were developed by Proteintech Group Inc, Rabbit CsgF antibodies were obtained from Prof. Scott Hultgren, Washington University, St. Louis, USA, Mouse His-tag antibodies were procured from ABGENT (catalog number: AM1010A), IRDye 800CW Goat anti-Rabbit IgG Secondary Antibody was purchased from LI-COR (catalog number: 926-32211), and Goat anti-Mouse IgG Secondary Antibody Alexa Fluor 568 was obtained from Invitrogen (catalog number: A-11004).

Validation

CsgA and CsgF antibodies are made by Proteintech Group Inc upon request. We have validated CsgA and CsgF antibodies by performing western blot on MC4100 bacterial wild-type cells and CsgA/ CsgF mutant cells. All other antibodies are commercially available and were validated by the manufactures.
